# Promoter-based identification of novel non-coding RNAs reveals the presence of dicistronic snoRNA-miRNA genes in *Arabidopsis thaliana*

**DOI:** 10.1186/s12864-015-2221-x

**Published:** 2015-11-25

**Authors:** Ge Qu, Katarzyna Kruszka, Patrycja Plewka, Shu-Yi Yang, Tzyy-Jen Chiou, Artur Jarmolowski, Zofia Szweykowska-Kulinska, Manuel Echeverria, Wojciech M. Karlowski

**Affiliations:** Department of Computational Biology, Institute of Molecular Biology and Biotechnology, Faculty of Biology, Adam Mickiewicz University in Poznan, Umultowska 89, 61-614 Poznan, Poland; Department of Gene Expression, Institute of Molecular Biology and Biotechnology, Faculty of Biology, Adam Mickiewicz University in Poznan, Umultowska 89, Poznan, 61-614 Poland; Agricultural Biotechnology Research Center, Academia Sinica, No. 128 Academia Rd. Sec. 2, Taipei, 115 Taiwan; Faculté des Sciences, Université de Perpignan via Domitia, 52, Av Paul Alduy, Perpignan, 66860 France

**Keywords:** *Telo*-box, Site II, non-coding RNA, snoRNA, miRNA, *cis*-regulatory elements

## Abstract

**Background:**

In the past few decades, non-coding RNAs (ncRNAs) have emerged as important regulators of gene expression in eukaryotes. Most studies of ncRNAs in plants have focused on the identification of silencing microRNAs (miRNAs) and small interfering RNAs (siRNAs). Another important family of ncRNAs that has been well characterized in plants is the small nucleolar RNAs (snoRNAs) and the related small Cajal body-specific RNAs (scaRNAs). Both target chemical modifications of ribosomal RNAs (rRNAs) and small nuclear RNAs (snRNAs). In plants, the snoRNA genes are organized in clusters, transcribed by RNA Pol II from a common promoter and subsequently processed into mature molecules. The promoter regions of snoRNA polycistronic genes in plants are highly enriched in two conserved *cis*-regulatory elements (CREs), *Telo*-box and Site II, which coordinate the expression of snoRNAs and ribosomal protein coding genes throughout the cell cycle.

**Results:**

In order to identify novel ncRNA genes, we have used the snoRNA Telo-box/Site II motifs combination as a functional promoter indicator to screen the Arabidopsis genome. The predictions generated by this process were tested by detailed exploration of available RNA-Seq and expression data sets and experimental validation. As a result, we have identified several snoRNAs, scaRNAs and 'orphan' snoRNAs. We also show evidence for 16 novel ncRNAs that lack similarity to any reported RNA family. Finally, we have identified two dicistronic genes encoding precursors that are processed to mature snoRNA and miRNA molecules. We discuss the evolutionary consequences of this result in the context of a tight link between snoRNAs and miRNAs in eukaryotes.

**Conclusions:**

We present an alternative computational approach for non-coding RNA detection. Instead of depending on sequence or structure similarity in the whole genome screenings, we have explored the properties of promoter regions of well-characterized ncRNAs. Interestingly, besides expected ncRNAs predictions we were also able to recover single precursor arrangement for snoRNA-miRNA. Accompanied by analyses performed on rice sequences, we conclude that such arrangement might have interesting functional and evolutionary consequences and discuss this result in the context of a tight link between snoRNAs and miRNAs in eukaryotes.

**Electronic supplementary material:**

The online version of this article (doi:10.1186/s12864-015-2221-x) contains supplementary material, which is available to authorized users.

## Background

The synthesis of functional transcripts in eukaryotes always involves non-coding RNAs (ncRNAs) within ribonucleoprotein complexes (RNPs) that direct the processing of larger precursor transcripts. Two major families of such molecules include snRNAs and snoRNAs, which direct the splicing and guide the chemical modification of RNAs, respectively [1]. In addition, the advent of complete genome sequencing coupled with high-throughput expression profiling technologies led to the discovery of thousands of novel ncRNAs in eukaryotes that regulate gene expression at nearly all levels. Among them, the most studied in the last decade have been the microRNA (miRNA) and small interfering RNA (siRNA) families, which direct gene silencing and chromatin condensation [2]. More recently, many longer ncRNAs (lncRNAs) with diverse sizes and structures have also been discovered and shown to play central roles in many biological processes [3], including regulation of the alternative splicing that controls flowering and other developmental processes in Arabidopsis [4, 5].

In the past decade, a major goal in plant biology research has been to identify novel ncRNAs that regulate cell growth, development or adaptation to biotic and abiotic stresses. Deep sequencing of size-fractionated RNAs has become a major source of ncRNA discovery, producing myriad ncRNA candidates [6]. The challenge remained, however, to distinguish functional ncRNAs from those produced by the transcriptional noise of the genome or from RNA degradation products. *In silico* approaches represent another important strategy for the identification of novel ncRNAs in a variety of sequenced genomes. Most of these techniques use algorithms that consider structural RNA features that are conserved in known RNA families. Here, we propose a distinct approach that employs conserved promoter elements of plant snoRNA genes for the identification of novel ncRNAs in the Arabidopsis genome.

The snoRNAs represent an abundant family of ncRNAs found in the nucleoli of all eukaryotes. Most snoRNAs belong to one of two subclasses, C/D box snoRNAs and H/ACA box snoRNAs, which guide 2’-O-ribose methylation and the pseudouridylation of specific RNA targets, respectively [7]. The C/D box and H/ACA box snoRNAs form two distinct conserved ribonucleoprotein (snoRNP) particles, characterized by fibrillarin (an RNA methylase) and dyskerin (Cbf5/NAP57; a pseudouridine synthase), respectively [7]. The C/D snoRNAs contain the conserved boxes C (RUGAUGA) near the 5' end and D (CUGA) near the 3’ end. Antisense elements complementary to the RNA target sequence are adjacent to the D box or an internal box, D’. The H/ACA snoRNAs generally form two hairpins connected by a hinge region, characterized by a conserved box, H (ANANNA), and an ACA trinucleotide motif located three residues upstream of the 3’ end. One or both of the hairpins contains an internal loop sequence that is complementary to flanking regions of their target uridine residue, which is modified to become pseudouridine.

Functionally similar to snoRNAs are scaRNAs, which direct RNA modifications of snRNAs in Cajal bodies. scaRNAs are larger than the predominant classes of snoRNAs and possess the characteristic boxes of both C/D and H/ACA snoRNAs as well as CAB boxes (UGAG), which function as Cajal body localization signals. Similar to snoRNAs, scaRNAs form conserved scaRNPs with fibrillarin and dyskerin [8].

snoRNAs have distinct targets and functions. Most are responsible for the modification of ribosomal RNAs (rRNAs), but others direct changes in other classes of RNAs, including tRNAs and snRNAs [7]. In addition, some essential snoRNAs such as the conserved U3 and U14 direct the specific endonucleolytic cleavage of ribosomal RNA precursors (pre-rRNAs) [7]. Moreover, many so-called ‘orphan’ snoRNAs have been described for which no target has been predicted, suggesting that they may have additional, currently unknown functions [9]. These snoRNAs have been found in humans and mice; compelling evidence has been gathered for a C/D snoRNA family that is expressed exclusively in the brain, where the snoRNAs target and control the alternative splicing of a serotonin receptor mRNA precursor [10, 11].

In addition to the canonical structured snoRNAs, many others have been discovered that, while maintaining the canonical C/D or H/ACA core structure, have additional extensions and fulfill extra functions. One example is the telomerase RNA (TR), a subunit of the RNP telomerase complex, which can guide the synthesis of telomeres in mammals. TR is a 400- to 500-nucleotide RNA characterized by a 3’ H/ACA snoRNA structure [12].

Furthermore, some snoRNAs have dual functions, as they are processed into small ncRNAs with miRNA-like functions in animals [13–15]. In plants, small RNAs derived from snoRNAs have been associated with AGO protein in both Arabidopsis and rice, but their role has not been elucidated [16, 17]. Additionally, some miRNA precursors have snoRNA features and can even function as snoRNAs [18, 19].

In Arabidopsis and rice, more than 200 canonical snoRNAs and scaRNAs have been identified so far [20–24]. Most plant snoRNAs are encoded by polycistronic genes for snoRNA precursors (pre-snoRNAs), which encode two or more snoRNAs. These pre-snoRNAs are released by endonucleolytic cleavage of the poly-snoRNA, and subsequent exonucleolytic trimming produces mature 5’ and 3’ snoRNA ends. In Arabidopsis, most polycistronic snoRNA genes are independent units that are transcribed by RNA pol II from a single promoter. However, some plant snoRNAs are also encoded within an intron of a protein-coding gene. These intronic snoRNAs, which can be either monocistronic or polycistronic, are released from the introns produced by pre-mRNA splicing. Notably, most of the host genes that encode intronic snoRNA in plants encode ribosomal protein genes (RP genes) or proteins related to ribosome biogenesis [25, 26].

Notably, in Arabidopsis, nearly all RP genes and other genes encoding proteins related to ribosome biogenesis and translation, together with polycistronic snoRNAs, share two conserved promoter elements: the *Telo*-box (AAACCCTA), which has a sequence related to telomere repeats, and the Site II element (TGGGCY) [27]. These two elements, hereafter called TeloSII, can be found in any orientation and order upstream of the promoter region containing the TATA box. Similarly, TeloSII elements characterize the promoters that control polycistronic snoRNAs and RP genes in rice [27]. An analogous situation occurs in yeast, in which the RP gene and snoRNA promoters share a characteristic telomere-related motif, aRCCCTaa, which is required for their transcription and is recognized by the telomere-binding protein Tbp1 [28]. Gain-of-function experiments in Arabidopsis showed that the *Telo*-box acts synergistically with the Site II element to coordinate the expression of these genes throughout the cell cycle [29]. Additionally, the Site II element has been demonstrated to bind to a transcription factor, TCP20, in Arabidopsis [30].

The overall data strongly indicate that TeloSII promoters coordinate the expression of snoRNAs along with that of protein-coding genes implicated in ribosome biogenesis and in translational control during the cell cycle. The polycistronic organization of snoRNAs would further contribute to the coordinated expression of these molecules in plants.

High-throughput sequencing approaches dedicated to the identification of novel functional ncRNAs produce an enormous amount of putative predictions; in plants, most predictions correspond to repeat-derived siRNAs originating from spurious transcription or RNA degradation. Likewise, *in silico* approaches designed to detect novel ncRNAs are mainly based on algorithms considering the structural features of known RNA families. Here, to reduce the noise-related 'RNA background' and to enhance the detection of new RNA families, we propose an approach based on using the TeloSII promoter motifs of the plant snoRNA genes to search for novel ncRNAs. Application of this strategy to the Arabidopsis genome resulted in the discovery of 26 novel snoRNA-like species, 16 novel ncRNAs without any relation to already described RNAs, and two dicistronic snoRNA-miRNA genes. We further show that this arrangement can also be found in rice, suggesting a tight evolutionary relationship between snoRNAs and microRNAs in plants.

## Results

### Computational approach to the identification of novel ncRNAs containing *Telo*-box and Site II regulatory elements

**Fig. 1:**
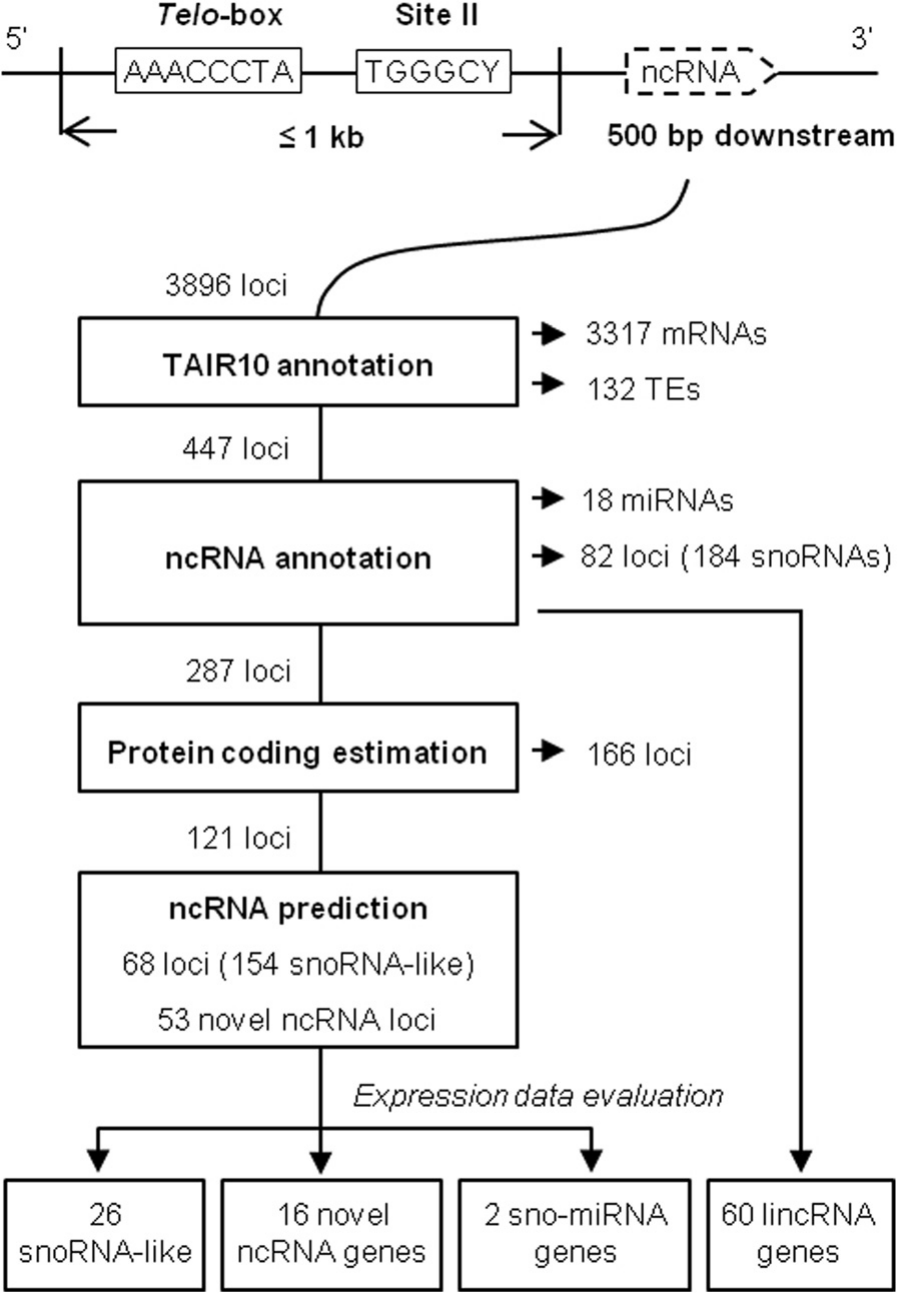
Computational analysis pipeline for the identification of ncRNA genes containing TeloSII elements in Arabidopsis

To systematically identify novel non-coding RNA (ncRNA) genes directed by TeloSII *cis*-regulatory elements in Arabidopsis, we assembled a customized pipeline (Fig. 1). We considered a region as a candidate promoter if both the *Telo*-box and Site II elements (TeloSII) were found (regardless of their order and distance) in a sequence window of 1 kb as suggested earlier by Gaspin et al. [27]. The next step in prediction involved testing adjacent regions. Based on the features of known snoRNAs obtained from previous studies [20–24], we scanned 500 nt downstream of TeloSII for existing gene annotations. For that purpose, we used annotations contained in the Arabidopsis TAIR10 database [31] and information contained in both the microRNA database (miRBase) [32] and the Plant Long ncRNA database (PLncDB) [33]. Finally, all predicted ncRNA candidates passing the above criteria were analyzed using Rfam, which reported the full collection of validated ncRNAs and their classification into distinct RNA families [34].

The first screening identified 3896 non-redundant TeloSII loci in Arabidopsis intergenic regions, of which 3317 loci were placed upstream of the transcription start sites (TSSs) of annotated protein coding genes (Additional file 1: Table S1). These included all of the RP gene promoter regions previously described by Gaspin et al. [27]. These mRNA encoding loci were not examined in subsequent steps. In addition, 132 loci contained signatures of transposable elements (TEs). Although there is strong evidence that functional ncRNAs, including several snoRNAs and miRNAs, are related to TEs in eukaryotes [35–37], we decided not to consider them for further analysis to maintain stringent conditions to identify the most promising candidates.

Among the remaining loci, 160 mapped to known ncRNA genes. These correspond to 82 loci encoding 184 known snoRNAs characterized in previous studies [20–24, 27] and 18 genes encoding miRNAs annotated in miRBase [32]. In addition, 60 TeloSII loci mapped to genes expressing long intergenic ncRNAs (lincRNAs) that were previously identified [38, 39] and recorded in PLncDB [33]. These results confirmed that our approach of using TeloSII as an indicator could effectively detect functional ncRNAs and allowed the identification of 287 potential novel ncRNA candidates.

In the next step, we estimated the protein-coding potential of the 287ncRNA candidates. To this end, we adopted ESTScan [40], a program used to detect protein coding regions in ESTs. This step revealed that 166 of the 287 candidate loci had protein coding capability. To maintain stringent parameters for the selection of ncRNAs candidates, we excluded these sequences from further analyses.

### Characterization of novel snoRNAs

The remaining 121 ncRNA candidates were analyzed using SnoReport, an algorithm that predicts both C/D and H/ACA box snoRNA/scaRNAs [41], and were subsequently processed with Rfam tools [34] to detect any RNA family signature in the remaining predictions. This process identified 68 TeloSII loci associated with snoRNA-like gene candidates (Fig. 1). A detailed survey of these genomic regions using SnoReport showed that most of these predictions corresponded to polycistronic genes encoding two or more snoRNAs, thereby corresponding well to the organization of plant snoRNA genes [25].

We further restricted our selection to those loci whose expression was supported by cDNA, EST, or RNA-Seq data available in the TAIR database. This restriction resulted in the designation of 12 polycistronic/monocistronic TeloSII gene loci for further detailed analyses (Table 1). The 12 TeloSII loci mapped onto ten polycistronic and two monocistronic snoRNA genes. Overall, these loci encode 26 novel snoRNAs, including three H/ACA box and 23 C/D box snoRNAs (Table 1). We noted that six of them (marked with triangles and asterisks in Table 1) had already been described as lincRNAs or intermediate-sized RNAs and shown to be expressed, albeit without functional assignment [38, 39].

**Table 1:** List of snoRNA/scaRNA candidates

ID^a^		Coordinates^b^	Type	Putative target	Copies (TeloSII)^c^	cDNA/EST	RNA-Seq	Tissue	Homology^d^
**Cluster1** ^△^	**ncR1**	Chr1:14283553..14283663	H/ACA	/	9(1)	/	GSM893120	flowers	*A.ly*
	**ncR2**	Chr1:14283835..14283886	C/D	/	4(1)	/	/	/	*/*
	**ncR3**	Chr1:14283975..14284049	C/D	18S/Am1750 (D)	5(1)	/	GSM442932	roots	*/*
	**ncR4**	Chr1:14284343..14284424	C/D	18S/Cm307 (D)	2(1)	/	GSM575247	flowers	*A.ly*
Cluster2	ncR5	Chr2:1836965..1837036	C/D	25S/Cm2509 (D)	1	/	GSM893122	flowers	*A.ly*
	ncR6	Chr2:1837308..1837401	C/D	18S/Gm275 (D)	1	/	GSM893120	flowers	*A.ly*
	ncR7	Chr2:1837461..1837557	C/D	U2.4/Gm331 (D)	12(1)	/	GSM893120	flowers	*A.ly*
Cluster3	ncR9	Chr2:5830603..5830668	C/D	U5/Tm687 (D)	2(1)	/	GSM893122	flowers	*Cru*
	ncR10	Chr2:5831355..5831456	C/D	U6.29/Cm590 (D)	4(1)	/	GSM893123	flowers	*A.ly*
Cluster4	ncR11	Chr4:1480138..1480215	C/D	25S/Cm2616 (D)	1	/	GSM893120	flowers	*A.ly, C.ru*
	ncR12	Chr4:1480326..1480415	C/D	U4.1/Am223 (D)	1	/	GSM893121	flowers	*A.ly*
**Cluster5**	**ncR13**	Chr5:3642290..3642331	C/D	U6.29/Am296 (D')	1	/	/	/	*/*
	**ncR14**	Chr5:3642496..3642628	C/D	U6.26/Um774 (D)	1	/	SRR505744	leaves	*A.ly, C.ru, B.ra*
Cluster6	ncR15	Chr5:20313629..20313716	C/D	/	1	/	GSM893122	flowers	*/*
	ncR16	Chr5:20313841..20313928	C/D	/	1	/	GSM893123	flowers	*A.ly*
	ncR17	Chr5:20314434..20314506	C/D	U5/Am921 (D)	1	/	GSM893121	flowers	*A.ly*
**Cluster7**	**ncR20**	Chr2:9809815..9809713	C/D	U6-29/Am21 (D)	1	EH971193	SRR505743	seedlings, roots	*A.ly*
	**ncR21**	Chr2:9809455..9809345	C/D	U2.4/Cm143 (D)	1	/	SRR505745	flowers	*A.ly, C.ru*
Cluster8	ncR22	Chr4:5918068..5917991	C/D	U2.3/Am345 (D)	8(6)	/	GSM893121	flowers	*A.ly*
	ncR23	Chr4:5917772..5917612	C/D	U2.9/Tm207 (D)	12(4)	/	GSM456944	flowers	*A.ly*
Cluster9	ncR24	Chr5:10673349..10673223	H/ACA	/	1	/	GSM893122	flowers	*A.ly*
	ncR25	Chr5:10672936..10672800	H/ACA	/	11(2)	/	GSM893121	flowers	*A.ly*
**Cluster10**	**ncR26**	Chr1:28889830..28889897	C/D	U6.1/Cm740 (D)	1	/	GSM896913	leaves	*A.ly, Cru, B.ra*
	**ncR27** ^△*^	Chr1:28889966..28890048	C/D	U6.1/Am177 (D)	1	/	GSM896913	flowers	*A.ly, Cru*
**Single**	**ncR18**	Chr1:26010695..26010627	C/D	18S/Am1558 (D)	1	/	/	/	*A.ly*
Single	ncR28^△^	Chr1:6140665..6140735	C/D	18S/Gm727 (D)	1	/	GSM893122	flowers	*/*

^a^ID with triangle was also identified as long ncRNA by Liu et al. [38]. ID with asterisk was also identified as intermediate ncRNA by Wang *et al.* [39]. ID marked in bold was verified by RT-PCR in this study

^b^Termini were predicted by snoReport program, and fine-turned based on small RNA enrichments and RT-PCR validation

^c^Numbers within parentheses denote copies also containing TeloSII elements

^d^Conservation analysis were performed in *Arabidopsis lyrata (A.ly), Capsella rubella (C.ru), Brassica rapa (B.ra), Medicago truncatula (M.tr)* and *Oryza sativa (O.sa)*

As in the case of most previously annotated Arabidopsis snoRNAs [20], multiple copies of ten of the predicted novel snoRNAs can be found in the Arabidopsis genome by using BLASTN (e-value < 1e-5; similarity > 80 %). However, not all of the snoRNA copies are associated with TeloSII elements. For example, there are 12 copies of cluster 2 in the genome, but only one copy has TeloSII elements (Table 1).

Comparative analysis with BLASTN and Phytozome database (version 10) [42] revealed that many of the predicted snoRNA sequences showed a high level of conservation among the closely related species *Arabidopsis lyrata*, *Capsella rubella* and *Brassica rapa* (Table 1). No similar sequences were identified in the genomes of more distant dicotyledonous (*Medicago truncatula)* or monocotyledonous (*Oryza sativa*) plant genomes. However, the absence of conservation in the distant species must be taken with caution. The plant snoRNAs exhibit varying degrees of divergence among species according to their targets. Some are well conserved among distant species like Arabidopsis and rice, while others are restricted to closely related ones. However, snoRNAs with different sequences can still be functional orthologs, targeting the same rRNA residue for modification in the two distant species [23, 25]. Alternatively, the sequence conservation between closely related species and lack of homologs in distant ones may indicate the recent origin of the identified novel snoRNA genes.

**Fig. 2:**
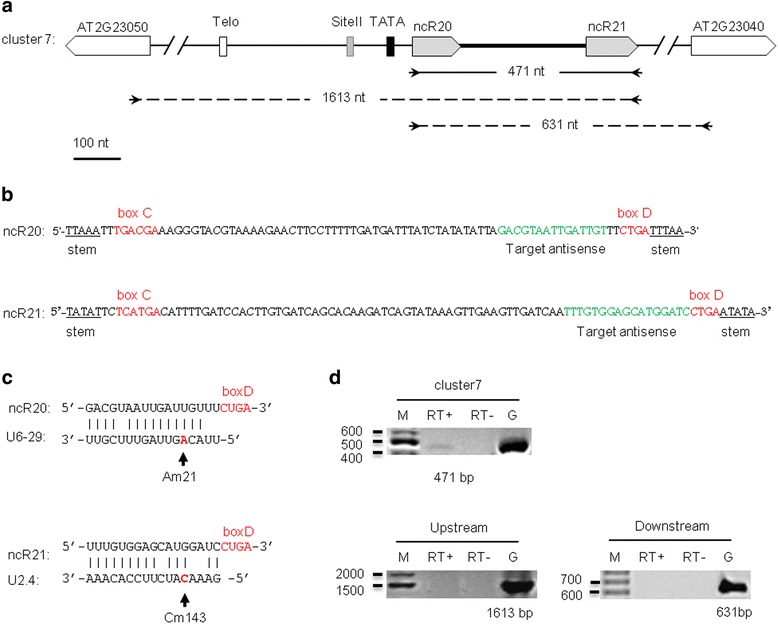
Identification of the clustered scaRNAs ncR20 and ncR21. (**a**) Schematic representation of scaRNA cluster 7. The scaRNAs are denoted by gray pentagons. The TeloSII and the TATA elements are represented by boxes: Telo (open box), Site II (gray box), and TATA (black box). The primers designed for cluster 7 and the expected amplicon sizes are indicated by arrows and a solid line. Additional primers corresponding to the adjacent genes designed for evaluation of independency of transcription of cluster 7 are shown as dashed lines. (**b**) The sequence features of ncR20 and ncR21: motifs C/D and the target antisense region are indicated in red and green colors, respectively; double-stranded-forming regions at the termini are underlined. (**c**) Predicted targets of ncR20 and ncR21. The positions of predicted methylated residues are indicated bold letters and red color. (**d**) RT-PCR analysis of cluster 7 precursor expression in 2-week-old seedlings. RT+ and RT- indicate the presence or absence of reverse transcriptase in the reaction, respectively. Molecular markers are shown in lane labeled with M. Genomic DNA (lane labeled with G) was used as positive control. The expected RT-PCR product sizes are provided below the lanes with product amplified on genomic DNA. The absence of amplification signals in “Upstream” and “Downstream” panels indicates the independent from neighboring genes character of cluster 7 transcription

Targets for C/D and H/ACA snoRNAs were predicted using the PLEXY [43] and RNAsnoop [44] programs, respectively. Seven snoRNAs were shown to target the methylation of 18S or 25S rRNA-specific residues (Table 1). In addition, 13 other predicted C/D box snoRNAs were found to target the spliceosomal snRNAs U2, U4, U5 and U6. Therefore, these should be considered as scaRNAs, as the modification of these snRNAs mainly occurs in Cajal bodies [8]. We estimated the minimal size of the predicted canonical snoRNA and scaRNA transcripts as the distance between the C and D boxes for the C/D snoRNAs and between the stem structure and the 3’ terminal ACANNN element for the H/ACA snoRNAs (Table 1). Figure 2 shows ncR20 and ncR21, two examples of canonical scaRNAs that are encoded by a dicistronic gene labeled as cluster 7. The expression of cluster 7 was evaluated in seedlings and shown to be specific for the predicted gene by RT-PCR (Fig. 2a and 2d). We have also predicted targets for the ncR20 and ncR21 to be U6-29 and U2.4 RNAs, respectively (Fig. 2c).

For the remaining three predicted C/D box and three predicted H/ACA box snoRNAs we could not find the target; therefore, following the widely accepted classification they should be rather considered as 'orphan' snoRNAs [9]. For these orphan snoRNAs, the transcript could be much longer than the predicted snoRNA structure, as there have been several previously described cases of non-canonical snoRNAs and scaRNAs with sequence extensions that have varying and important biological functions (see Discussion).

In most cases, expression of the predicted snoRNAs/scaRNAs is supported by RNA-Seq data. However, except for ncR20, which is encoded by cluster 7 (Table 1), none has any associated EST or cDNA. Notably, this is also the case for other predicted snoRNAs that were previously reported as lincRNAs but that have been experimentally confirmed to be transcribed [38, 39]. The absence of EST sequences is likely due to the lack of polyA tails on the eukaryotic snoRNAs. In yeast transcription, the termination of snoRNA genes by RNA Pol II utilizes a distinct mechanism that is not associated with polyadenylation [45]. In plants, snoRNAs encoded by polycistronic genes are produced by endonucleolytic cleavage of the precursors and subsequent exonucleolytic trimming of the released snoRNAs to generate their mature extremities [25, 26].

Cluster 1 was previously reported to be expressed as individual snoRNAs, represented by independent lincRNAs, rather than as a polycistronic transcript. To confirm our prediction of the clustered expression of these snoRNAs, we amplified the polycistronic precursor by RT-PCR. Our result clearly shows the amplification of one precursor encoding predicted snoRNAs (Additional file 2: Figure S1). A similar result was observed for cluster 10, showing amplification of the predicted dicistronic precursor.

We also confirmed the expression of the predicted snoRNA gene loci encoding ncR18, for which no evidence of expression was available in the expression databases, by RT-PCR. The result clearly shows the specific amplification product of an ncR18 transcript, confirming its expression in 2-week-old seedlings (Additional file 2: Figure S1).

In summary, the evidence presented here reveals 26 novel snoRNA-like candidates that are regulated by TeloSII elements. Seven C/D snoRNAs are predicted to target rRNA for methylation, 13 represent scaRNAs and could modify snRNAs, and six have characteristics of orphan snoRNAs and probably fulfill additional, unknown functions.

### A novel class of polycistronic sno-miRNA genes

Notably, three TeloSII loci were mapped to predicted snoRNA genes that are encoded in the proximity to annotated three distinct miRNA loci: miR775, miR779 and miR158b (Additional file 3: Table S2). Because we could not find evidence that the predicted snoRNA associated with miR158b was expressed (data not shown), we focused on analyzing the novel dicistronic genes snoRNA-miR775 and snoRNA-miR779.

Arabidopsis miR775 and miR779 are non-conserved miRNAs that were identified by the deep sequencing of small RNA libraries from*rdr2* mutant plants [46]. Their expression was shown to be very low but was confirmed by northern blot hybridization using LNA oligonucleotide probes. Additionally, the biogenesis of both miRNAs was shown to be specifically dependent on the DCL1 enzyme [46].

**Fig. 3:**
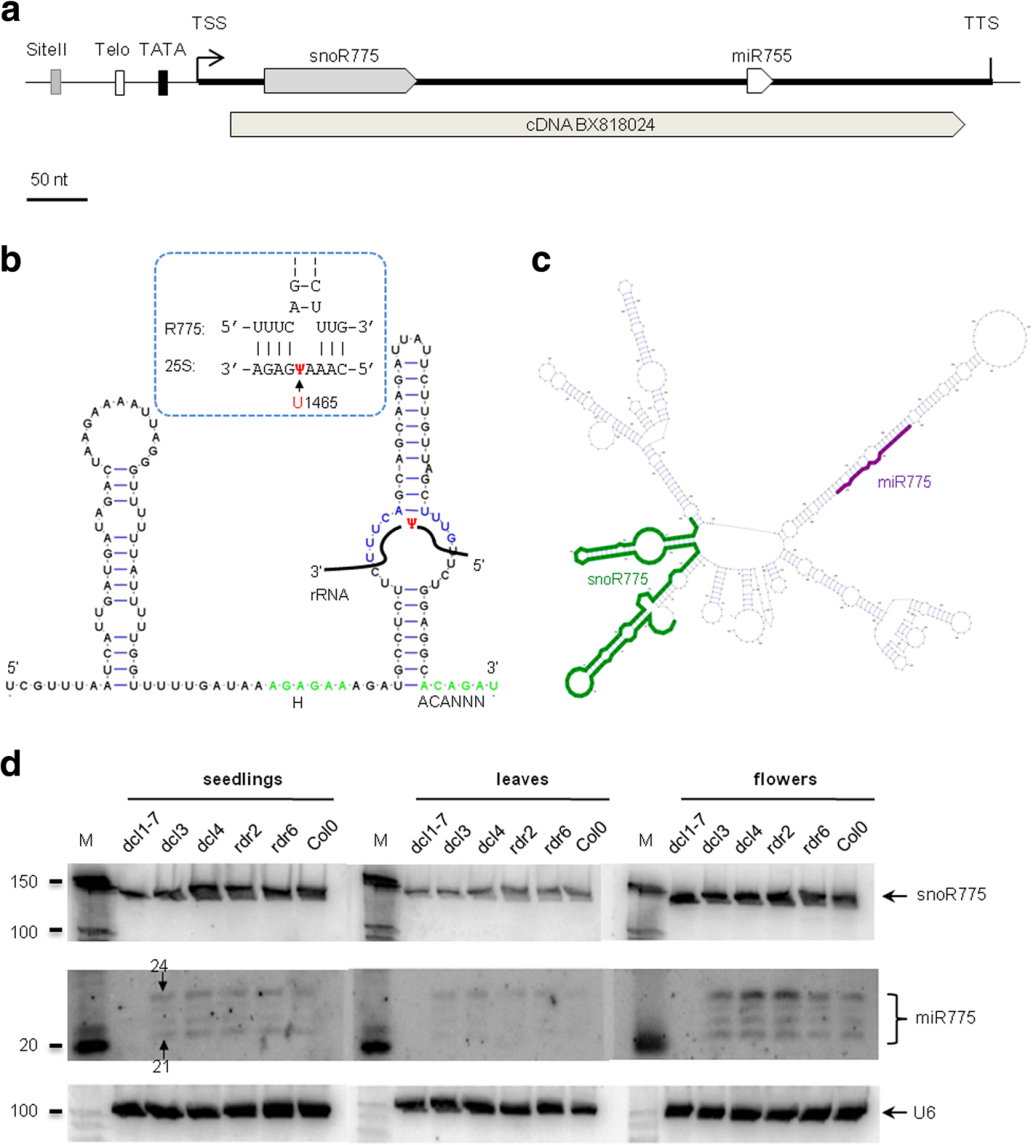
Schematic representation and experimental validation of the sno-miR775 gene. (**a**) Gene organization of sno-miR775. snoR775 and miR775 are denoted by a gray pentagon and an open pentagon, respectively. The TeloSII elements and TATA box are represented by different boxes: Telo-box (open box), Site II (gray box), and TATA box (black box). Both snoR775 and pre-miR775 are covered by cDNA BX818024. The transcription start site (TSS) and transcription termination site (TTS) were determined by 5’ RACE and 3’ RACE, respectively. (**b**) The centroid secondary structure of snoR775, drawn by using the RNAfold program. The H and ACA boxes are denoted by green color. The complementary region to the predicted target 25S rRNA is represented by blue color, and details are shown in the blue dashed box. Ψ: pseudouridylation site. (**c**) Secondary structure of the sno-miR775 precursor sequence. Mature miR775 and the predicted snoRNA are in purple and green colors, respectively. (**d**) Northern blot analysis of sno-miR775. The hybridization was carried out in 3 different tissues: 2-wk-old seedlings, 3-wk-old leaves and 5-wk-old flowers. Lanes in the blots represent the following samples: *dcl1-7*, *dcl3*, *dcl4*, *rdr2*, and *rdr6*as well as wild-type Col0. U6 was used as the loading control

The region upstream of the predicted miR775 precursor (pre-miR775) contains a classical TeloSII motif associated with a TATA box element (Fig. 3a). Screening of the flanking genomic sequences with the SnoReport program revealed a sequence that can fold into a typical H/ACA box snoRNA structure, including two stems separated by the conserved ANANAA motif, a 3’ terminal ACANNN motif, and an internal loop sequence that could direct the pseudouridylation of the 25S rRNA U1465 residue (Fig. 3b). We confirmed the expression of the mature 150-nt H/ACA snoRNA by northern blotting in seedlings, leaves and flowers (Fig. 3d). Based on the close proximity of this snoRNA to miR775, we named it snoR775.

Several lines of evidence indicate that miR775 and snoR775 are co-transcribed and produced from the same precursor. The first line of evidence is the presence of a 605 nt cDNA (BX818024) that encompasses both snoR775 and pre-miR775 (Fig. 3a). The single-transcript organization was confirmed by RT-PCR using a pair of primers encompassing snoR775 and miR775 (data not shown). Finally, we mapped the transcription start site (TSS) and transcription termination site (TTS) using 5’ RACE and 3’ RACE, respectively (Fig. 3a). A single major signal clearly mapped the TSS upstream of snoR775, whereas the TTS was mapped downstream of pre-miR775 (Additional file 4: Figure S2). In conclusion, the snoR775 and miR775 precursors are encoded by a single gene that is transcribed as a 655-nt dicistronic transcript (Fig. 3c). We named this gene sno-miR775. More details about the small RNA sequencing data corresponding to sno-miR775 are shown in Additional file 5: Figure S3a.

The identification of this transcript raised a question regarding how snoR775 and miR775 are produced from the same precursor, considering that each RNA uses a different biogenesis pathway. The processing of pre-miRNA depends on the endonuclease DCL1, whereas the processing of polycistronic pre-snoRNAs is initiated by the RNase III homolog of the yeast Rnt1 enzyme [47]. We tested the effect of different mutations in the genes that control miRNA and siRNA biogenesis on the expression of snoR775 and miR775. The result clearly shows that the expression of snoR775 is not affected in any of these mutants, whereas the expression of miR775 specifically depends on DCL1 (Fig. 3d), as previously reported [46]. This result indicates that the biogenesis of snoR775 produced from the sno-miR775 dicistronic precursor is independent from the miR775 biogenesis pathway.

The presence of four miR775 signals ranging from 21 to 24 nt (Fig. 3d) is puzzling but does not represent a unique case in plants. In addition to the canonical 21 nucleotides miRNAs produced by DCL1, this enzyme has been reported to produce longer miRNAs up to 24 nucleotides. Most notably in the case of miR173, miR472 and miR828, which make for both 21 and 22 nucleotides miRNAs, it was clearly shown that the production of 22 nucleotides miRNAs depends on the nature of the foldback structure of the pri-miRNA. Significantly, these 22 nucleotides miRNAs were shown to have important roles in driving RDR6-dependent siRNA biogenesis [48]. One possibility is therefore that the presence of a snoRNA within the pri-miR775 alters somehow the canonical precursor structure processed by DCL1 and induces production of the additional 22 to 24 nucleotides miR775 fragments. Alternatively, one can also imagine that processing of the snoRNAs, which implies previous assembly of a RNP complex on the snoRNA precursor [49] could interfere with DCL1 accuracy in the maturation of the miR775. Another possibility that could explain the presence of the longer miR775 species may involve modification of the mature miRNA molecule by addition of extra residues (e.g., uracil) [50].

**Fig. 4:**
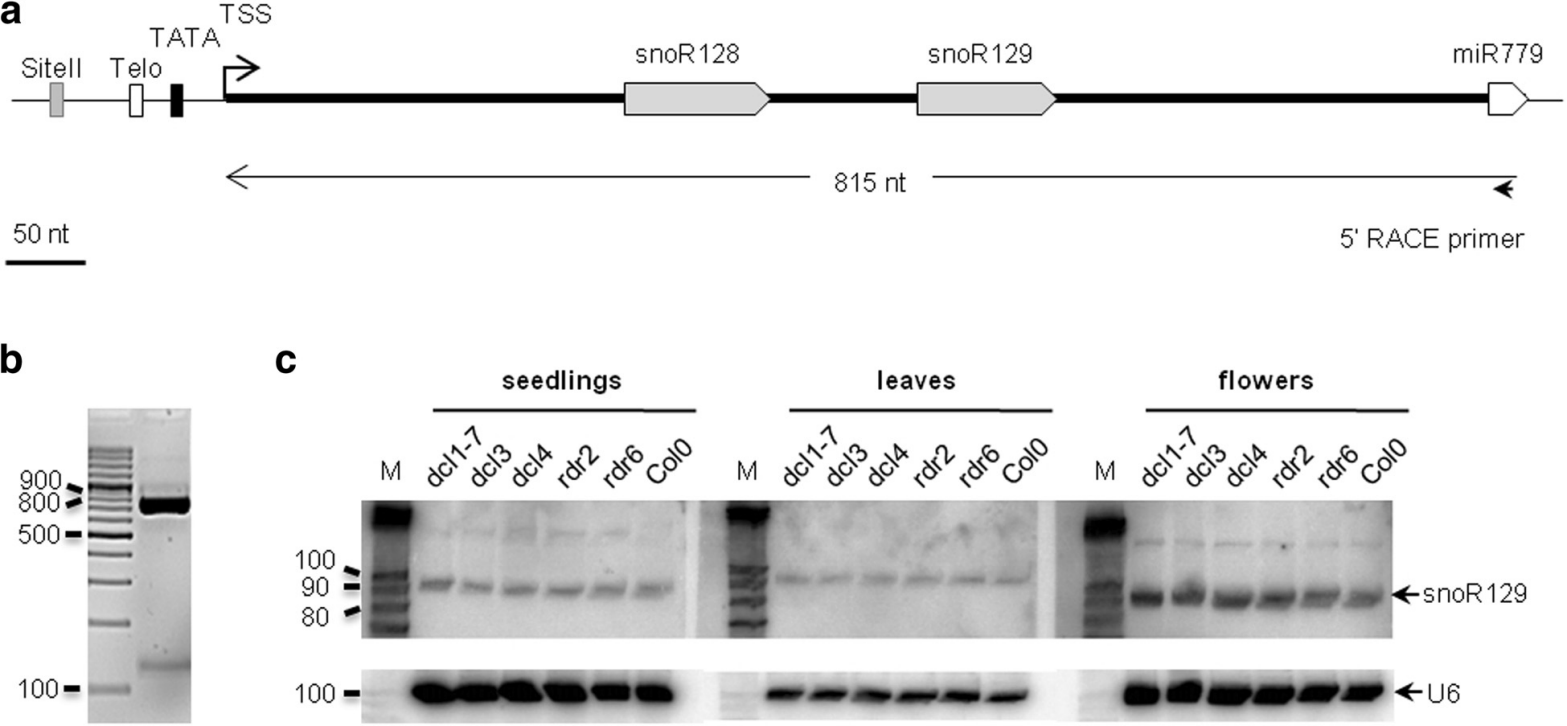
Schematic representation and experimental validation of the sno-miR779 gene. (**a**) Gene organization of sno-miR779. The snoRNAs and miR779 are denoted by gray pentagon and open pentagon, respectively. The TeloSII elements and TATA box are represented by different boxes: Telo-box (open box), Site II (gray box), and TATA box (black box). The transcription start site (TSS) was determined by 5’ RACE. (**b**) 5’ RACE amplification of snoR779. A single major signal in 5’ RACE PCR indicates the transcription start site (TSS); the product size, confirmed by sequencing, was 815 nt. (**c**) Northern blot analysis of snoR129. The hybridization was carried out in 3 different tissues: 2-wk-old seedlings, 3-wk-old leaves and 5-wk-old flowers. The lines in the blots include the following samples: *dcl1-7*, *dcl3*, *dcl4*, *rdr2*, and *rdr6* mutants and wild type Col0. U6 was used as the loading control

A similar genomic organization was observed for the sno-miR779 gene (Fig. 4a). The TeloSII elements are located upstream of a dicistronic snoRNA gene cluster encoding C/D box snoR128 and snoR129. These two snoRNAs were previously identified and shown by northern blot to accumulate in vivo [20, 22].

No cDNA sequence representing this locus could be found. Therefore, to confirm that the two snoRNAs and the miR779 precursor are co-transcribed, we mapped the transcription start site by 5’ RACE with a primer complementary to 3’ end of pre-miR779. The result showed a major signal mapping upstream of the snoR128 that encompassed snoR128, snoR129 and the pre-miR779 precursor (Fig. 4a & 4b). Furthermore, northern blot analysis indicated that the expression of snoR129 was not affected in miRNA and siRNA mutant lines (Fig. 4c). However, in the northern blot analysis, we could not detect the expression of miR779, most likely because of the very low expression level of this miRNA, as previously reported [46]. Like snoR775, the expression of snoR129 is independent of DCL1; however, DCL1 is required for the expression of miR779 [46]. More details about the small RNA sequencing data corresponding to sno-miR779 were shown in Additional file 5: Figure S3b.

The identification of these two sno-miRNA genes in Arabidopsis led us to search for a similar arrangement in the rice genome, where TeloSII elements have also been shown to be conserved upstream of RP genes and snoRNA genes [27]. We screened the genomic regions flanking the annotated rice miRNAs with the SnoReport program. This approach identified 20 sno-miRNA candidates, four of which were predicted to be controlled by TeloSII elements (Additional file 6: Table S3). Additionally, two of the predicted snoRNA-miRNA precursors, corresponding to miR1850 and miR6250, were adjacent to canonical polycistronic snoRNA genes that had been previously reported [17, 24] (Additional file 7: Figure S4a).

osa-miR1850, which has been reported by several groups, has been found to be associated with the rice protein AGO1, and its target transcript (Os04g47410) has been experimentally validated [51]. The miR1850 genomic locus is located immediately downstream of a snoRNA cluster encoding four different snoRNAs that have been shown to be expressed [24]. Upstream of this locus, we detected the TeloSII TATA motif (Additional file 7: Figure S4a). The rice inflorescence RNA RT-PCR assay, used to amplify the predicted common precursor by using primers surrounding the three snoRNAs and miR1850, did not produce a detectable product, suggesting that the snoRNA and miRNA transcripts are processed independently. However, more detailed analysis points towards a more complex picture. As previously shown, the miR1850 transcript is processed into two splicing isoforms [52]. One has a single exon, and the second has an intron encapsulating the snoRNA-miRNA cluster. Therefore, the primers designed for the expression study targeted the intron of the second splicing variant (Additional file 7: Figure S4a). Assuming that the regulation and processing of non-coding RNA introns are complex, we suppose that the absence of an RT-PCR signal (Additional file 7: Figure S4b) could be due to the differences in abundance of the two splicing variants in the tested tissues.

miR6250 has been identified by high-throughput sequencing of small RNA fractions and reported to be highly expressed in rice roots [53]. The predicted miR6250 gene structure also overlaps with a previously described polycistronic snoRNA cluster (Additional file 7: Figure S4a) [17]. This locus is preceded by a Site II element and a TATA box but has no detectable *Telo*-box motif. The existence of a cDNA sequence (AK107197) encompassing the whole region suggests that the snoRNAs and miR6250 are co-transcribed and are probably derived from the processing of a common precursor. The presence of single transcript representing the shared precursor was also confirmed by RT-PCR (Additional file 7: Figure S4b). However, notably, the sequence encoding the precursor of osa-miR6250 [17] overlaps with the sequence of osa-snoR111 (Additional file 7: Figure S4a), an H/ACA box snoRNA that is conserved in plants [17]. It has been previously reported by Liu et al. [17] that some small RNAs in range of 20–30 nt and associated with AGO proteins could be derived from snoRNA precursors in rice. Our analysis indicates that both snoR111 and miR6250 overlap, therefore it seems that osa-miRNA is likely to be one such case.

The examples of miR6250 and miR1850 most likely reflect the evolutionary relationship between snoRNAs and miRNAs (see Discussion). To further investigate the relationship between snoRNAs and miRNAs, we screened the flanking regions of each annotated rice miRNA recorded in miRBase to search for closely located snoRNA signatures by SnoReport program, independent of the presence of TeloSII elements. Based on the observation of the average size of snoRNA precursors achieved from previous studies [20–24], we set 500 nt as the window size of flanking regions. If any putative snoRNA can be predicted and not overlapped with mature miRNA in the flanking regions, it would be considered as a snoRNA-miRNA candidate. This analysis revealed several examples of miRNA precursors that encompassed predicted snoRNAs and that were additionally supported by cDNA sequences (Additional file 8: Figure S5). However, further experimental studies must be conducted to confirm the presence of mature snoRNA molecules.

### Identification of novel ncRNAs

**Fig. 5:**
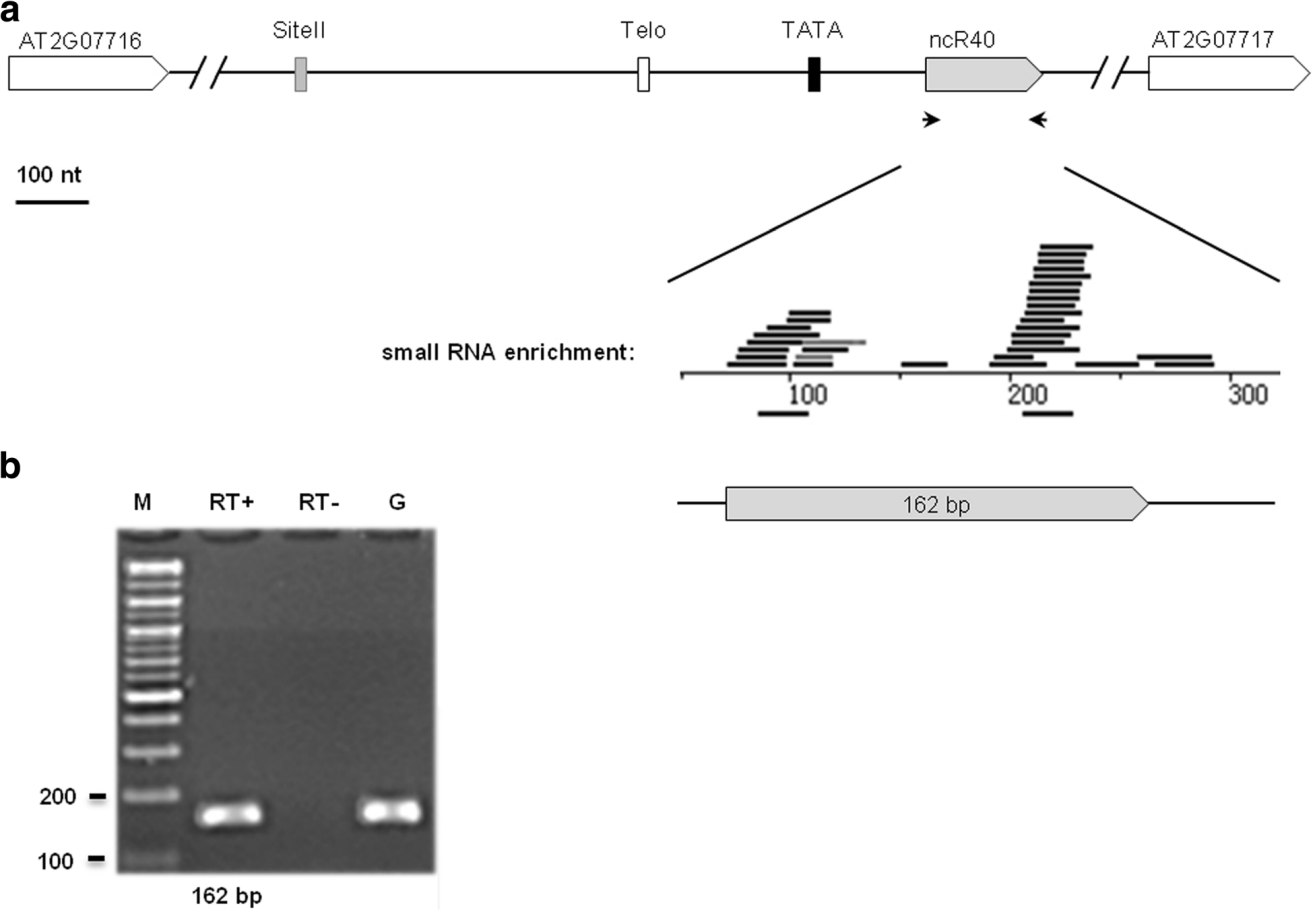
The identification of ncR40. (**a**) Schematic representation of ncR40. The termini were examined by small RNA fragments enrichment. The TeloSII elements and TATA box are represented by different boxes: Telo-box (open box), Site II (gray box), and TATA box (black box). The primers designed for ncR40 and their expected sizes are indicated by arrows and solid lines. (**b**) RT-PCR validation of ncR40 in 2-week-old seedlings. RT+ and RT- refer to the presence and absence of reverse transcriptase, respectively. Molecular markers are shown as M. Genomic DNA, G, was used as a positive control. The expected RT-PCR product size is indicated below the lanes

The 53 remaining ncRNAs located downstream of TeloSII elements (Fig. 1) did not show similarity to any known RNA family reported in the Rfam database. Among these ncRNAs, 16 loci were supported by RNA-Seq or MPSS signatures and/or corresponding ESTs or cDNAs, but had no predicted protein-coding capabilities (Table 2). The presence of corresponding MPSS/EST/cDNA sequences indicates that, in contrast to the identified snoRNAs (Table 1), these ncRNAs are polyadenylated.ncR40 is an interesting example; its expression is supported by short reads from RNA-Seq and RT-PCR experiments (Fig. 5). Another seven RT-PCR-validated ncRNAs are shown in Additional file 9: Figure S6.

**Table 2:** List of novel “Telo + site II” ncRNAs

ID^a^	Coordinates^b^	Strand	Copies^c^	EST/MPSS	RNA-Seq	Tissue	Homology^d^
**ncR30**	Chr1:471418..471193	reverse	1	EL289958	SRR420813	seedlings, roots	*A.ly, C.ru*
**ncR33**	Chr1:6700408..6700704	forward	2(1)	EL057995	GSM800621	seedlings, roots	*A.ly, C.ru, B.ra*
ncR34	Chr1:9925561..9925850	forward	1	EL068915	GSM893120	seedlings, flowers	*A.ly*
ncR36	Chr1:29342637..29342731	forward	2(2)	EL217991	GSM800621	seedlings	*A.ly*
**ncR40**	Chr2:3416235..3416396	forward	2(2)	EG424594	GSM893120	mixtures, flowers	*A.ly, C.ru, B.ra*
ncR41	Chr2:8932605..8932664	forward	1	EH901141	GSM881679	seedlings, leaves	*A.ly*
ncR42	Chr2:10463806..10464094	forward	1	EG501397	SRR505745	mixtures, flowers	*A.ly, B.ra*
**ncR43**	Chr2:14879905..14880181	forward	2(2)	EL282638	GSM800621	seedlings, roots	*A.ly*
**ncR45**	Chr3:1429026..1429094	forward	1	EH795486	/	seedlings, roots	*A.ly*
ncR46	Chr3:6000458..6000378	reverse	1	EL131833	/	seedlings	*A.ly*
ncR47	Chr3:8900985..8901049	forward	1	EH809410	SRR505744	seedlings, leaves	*A.ly*
**ncR49**	Chr3:19219613..19219701	forward	2(1)	EL055437	GSM869251	seedlings, flowers	*A.ly, B.ra*
ncR50	Chr4:9948779..9948834	forward	1	EL266852	GSM385393	seedlings, siliques	*A.ly, C.ru, B.ra*
**ncR51**	Chr4:13350013..13350417	forward	1	EH846374	SRR1184187	seedlings	*A.ly, C.ru, Bra*
**ncR52**	Chr5:3784963..3784812	reverse	1	EH955479	SRR420815	seedlings, roots	*/*
ncR53	Chr5:9386367..9386269	reverse	4(2)	EL145385	GSM575247	seedlings, flowers	*A.ly*

^a^ID shown in bold was verified by RT-PCR in this study

^b^Termini estimated based on deep sequencing data, along with cDNA/EST/MPSS sequences

^c^Numbers within parentheses denote copies also containing *Telo*-box/site II elements

^d^Conservation anA.lysis were performed in *Arabidopsis lyrata (A.ly), Capsella rubella (C.ru), Brassica rapa (B.ra), Medicago truncatula (M.tr)* and *Oryza sativa (O.sa)*

We also identified 60 lincRNAs located downstream of TeloSII elements (Additional file 10: Table S4). These RNAs were among the 6480 lincRNAs reported by Liu et al. [38] for which no function or structural similarity has been found. Using the Rfam database, we were able to identify 12 of these RNAs as snoRNAs and 1 as plant SRP RNA, which is the subunit of the Signal Recognition Particle RNP that is involved in protein translocation to the endoplasmic reticulum (Additional file 10: Table S4).

In summary, we have identified 60 non-coding RNAs (including 45 lincRNAs without any similarities to known RNAs; Additional file 10: Table S4) whose transcription seems to be under the control of TeloSII regulatory elements.

## Discussion

We have successfully identified functional ncRNAs by whole genome screening for TeloSII motifs. The RNAs identified in this study can be divided into three distinct groups.

### snoRNAs and scaRNAs

The first group includes 26 snoRNAs or scaRNAs encoded by ten polycistronic clusters and two monocistronic genes. Seven of these ncRNAs represent canonical C/D snoRNAs that target rRNA for methylation, adding previously unidentified members to the numerous snoRNA families that have already been found in Arabidopsis. Thirteen of the predicted molecules represent canonical scaRNAs that are predicted to target snRNAs. This result considerably increases the number of Arabidopsis scaRNAs because only 11 have been reported previously [22, 23, 49]. Finally, the remaining six predicted molecules represent orphan snoRNAs for which no RNA target could be found (Table 1). Notably, in the case of orphan snoRNAs, the transcripts may be much longer than the minimal size predicted by the SnoReport program, estimated as the distance between the conserved elements of canonical snoRNAs. For example, in the case of the telomerase RNA in mammals, the transcript is up to 450 nucleotides in length and has an H/ACA snoRNA structure at the 3’ end [12]. As another example, the C/D box scaR102 gene in Arabidopsis directs the transcription of a 370-nucleotide transcript that accumulates in seedlings and further generates mature scaR102, which is170 nucleotides long [49]*.*

### sno-miRNA genes

We have identified sno-miRNA genes encoding dicistronic precursors that are processed into both mature snoRNA and miRNA in eukaryotes. Previous reports have revealed numerous small RNA fragments that are derived from H/ACA and C/D box snoRNAs. However, fragments derived from snoRNAs have been shown to function as miRNAs in only a few cases, including humans and the protozoan parasite *Giardia lamblia* [13–15]. Notably, in Arabidopsis and rice, snoRNA-derived fragments have also been shown to be associated with AGO proteins, which are effectors of RISC complexes [16, 17]. However, the Arabidopsis dicistronic snoR-miR775 and sno-miR779 genes identified in this study represent a different case, as both the snoRNAs and the miRNA are processed from a common precursor using two distinct pathways that preserve the integrity of both ncRNAs (Figs. 3 & 4). This situation is similar to that of dicistronict snoRNA genes, which are specific to plants and produce mature tRNA and snoRNA molecules [54]. In addition, at least two similar dicistronic snoRNA-miRNA genes have been identified in rice: sno-miR1850 and sno-miR6250. However, as previously mentioned in the case of sno-miR6250, the miRNA is probably a snoRNA-derived fragment that is produced by processing conserved snoR111 (see Results).

A set of miRNA precursors has been shown to display snoRNA-like features or, in some cases, even to function as snoRNAs [19]. However, in all described examples for each precursor, despite their features, only one mature product (either snoRNA or miRNA) could be detected. We screened the annotated Arabidopsis miRNA loci to search for additional snoRNA-miRNA genes, without requiring the presence of TeloSII elements. This analysis revealed five additional loci that encoded potential snoRNAs associated with miRNAs (Additional file 11: Table S5). A similar analysis in rice revealed additional putative snoRNA-miRNA dicistronic genes (Additional file 6: Table S4 & Additional file 7: Figure S4). Although we do not know whether all these predicted snoRNAs are expressed from these loci, as we have shown for snoR775, snoR779 in Arabidopsis and the snoRNA cluster from the rice miR6250 genes, these additional data suggest an evolutionary relation of miRNAs and snoRNAs [19] most probably by mechanism similar to miRNA precursor formation [55], involving duplication of miRNA into an existing snoRNA precursors.

The snoRNAs, which are also present in Archaea, have ancient origins that can be traced back to a primary ancestor over 2 ~ 3 billion years ago. The snoRNA gene family was subsequently greatly expanded by retrotransposition associated with transposable elements (TEs) [19, 37] and by massive gene and chromosomal duplications [20]. This dramatic increase in the size of the snoRNA family was associated with diversification and the acquisition of new targets and functions.

miRNAs appeared later in evolution and are not present in Archaea, but their progression has also been related to TE expansion [35]. Interestingly, it has been proposed that during the process of snoRNA expansion and diversification, a subgroup progressively lost snoRNA functionality and gained new, possibly miRNA-related, capabilities [19]. This hypothesis would explain the association of many predicted snoRNAs with miRNA loci in Arabidopsis and rice presented in this study.

### ncRNAs with unknown functions

We have identified 60 ncRNAs (including 45 lincRNAs without similarities to any RNA signature; Additional file 10: Table S4) that are probably under the control of TeloSII regulatory elements. These elements have been shown to act synergistically to coordinate the expression of protein-coding genes related to ribosome biogenesis throughout the cell cycle in Arabidopsis [29, 30]. Although the function of these ncRNAs is not known, we can speculate that they play important cellular functions that must be coordinated throughout the cell cycle and could, as can all of the other proteins and snoRNAs, control ribosomal functions.

## Conclusions

We show that sequences of *Telo*-box and Site II regulatory elements, characteristic for promoters of genes encoding ribosomal proteins and polycistronic snoRNAs, can be successfully used for whole genome screening for novel non-coding RNAs. As expected, the large group of new predictions included novel snoRNAs and scaRNAs. In addition, we have found genes encoding RNA precursors that are processed into both mature snoRNA and miRNA. However, the largest collection of novel transcripts contained ncRNAs with unknown functions, providing inspiring opportunities for further research.

## Methods

### Plant material and growth conditions

Arabidopsis ecotype Columbia-0 wild type and *dcl1-7*, *dcl3-1*, *dcl4-2*, *rdr2-2*, and *rdr6-15* mutants were used throughout the study. Inflorescence tissue and leaves were harvested from plants grown in ‘Jiffy-7 42 mm’ soil spots (Jiffy International AS, Stange, Norway) in a growth chamber with a 16 h day (150–200 μmol/m/s), a constant temperature of 22°C and a humidity of 70 %. Seedlings were cultivated in 1/2 MS medium under the same conditions as above.

### DNA and RNA isolation

Genomic DNA was extracted from 2-week-old wild-type seedlings using the DNeasy Plant Mini Kit (Qiagen, Hilden, Germany). Total RNA was isolated from 2-week-old seedlings, 3-week-old rosette leaves, and 5-week-old inflorescence tissue using TRIzol reagent (Invitrogen, Life Technologies, USA). The RNA concentration was measured using a NanoDrop ND-1000 (NanoDrop Technologies, Wilmington, USA). DNA contamination was removed by digestion using a TURBO DNA-free kit (Ambion, Life Technologies, USA).

### Reverse transcription and PCR

Reverse transcription (RT) was performed using 3 μg of DNase-treated RNA, an oligo(dT) 18 primer (Novazyme, Poland) and SuperScript III Reverse Transcriptase (Invitrogen, Life Technologies, USA) according to the manufacturers’ instructions. cDNA samples were diluted 5 times, and 2 μl was used as template. In parallel, two additional reactions were carried out without SuperScript RT: one with DNase-treated RNA as a negative control and the other one with genomic DNA (from 2-week-old seedlings) as a positive control. Touch-down PCR amplifications were performed to detect promising candidate ncRNAs using the following thermal profile: 1 cycle of denaturation at 94 °C/1 min, annealing at 65°C/30 s, and elongation at 72 °C/1 min; 29 cycles of denaturation at 94 °C/30 s, annealing at 63 °C/30 s (∆ -0.5 °C/cycle), and elongation at 72°C/1 min; and an additional 10 ~ 13 cycles, depending on the expression level of the gene of interest, of denaturation at 94 °C/30 s, annealing at 53 °C/30 s, and elongation 72 °C/1 min. PCR products were visualized on 1x TBE/2 % agarose gels with 100 bp Plus and 1 kb Plus DNA Ladders (Thermo Fisher Scientific, Lithuania) as size markers. Primer sequences can be found in Additional file 12: Table S6.

### 5’ and 3’ RACE

To examine the termini of transcripts, 5’ and 3’ two-step RACE experiments were conducted with a SMARTer RACE cDNA Amplification Kit (Clontech, Mountain View, USA) according to the manufacturer’s protocol. PCR reactions were carried out using the Advantage 2 PCR Enzyme System (Clontech, Mountain View, USA). PCR products were cloned into the pGEM T-Easy vector (Promega, Madison, USA) and sequenced (Faculty’s Laboratory of Molecular Biology Techniques, Adam Mickiewicz University in Poznan, Poland). The primers are listed in Additional file 12: Table S6.

### Northern blot analysis of dicistronic snoRNA-miRNA genes

A total of 30 μg of RNA was separated by denaturing 8 M urea PAGE (15 %). RNA electrophoresis, blot transfer, and hybridization were performed as previously reported [56]. DNA oligo probes (Sigma) were 5’ labeled with [γ-32P]ATP (6000 Cimmol–1; Hartmann Analytic GmbH, Germany). Mature miRNA and snoRNA were detected on the same blot. The U6 hybridization signal was taken as a loading control. The Decade™ Markers System (Catalog Number: AM7778) was used as a size marker. The blots were exposed for 5 days to a phosphor imaging screen (Fujifilm) and scanned with a Fujifilm FLA5100 reader (Fujifilm Co., Ltd, Japan). Blots were quantified with Multi Gauge V2.2 software. Probe sequences are listed in Additional file 12: Table S6.

### Public data resources

Annotations for *Arabidopsis thaliana* (including mRNAs, TE fragments, cDNAs, ESTs and T-DNA insertion sites) and *Oryza sativa* were retrieved from TAIR (version 10, http://www.arabidopsis.org/) [31] and RGAP (version 7, http://rice.plantbiology.msu.edu/) [57], respectively. miRNA data were obtained from miRBase (Release 20, http://www.mirbase.org/) [32]. Known snoRNA sequences were collected from previous studies [20–24, 27]. LincRNAs verified by RNA-Seq were downloaded from PLncDB (http://chualab.rockefeller.edu/gbrowse2/homepage.html) [33]. Genome sequences from *Arabidopsis lyrata*, *Capsella rubella*, *Brassica rapa*, *Medicagotruncatula*and *Oryza sativa* were obtained from Phytozome (version 10, http://www.phytozome.net/) [42]. Ribosomal RNA sequences (25S rRNA, 5.8S rRNA and 18S rRNA) were retrieved from GenBank (http://www.ncbi.nlm.nih.gov/genbank/), and experimentally identified spliceosomal small nuclear RNA sequences were obtained from ASRG (http://www.plantgdb.org/SRGD/ASRG/) [58]. rRNA sequences, along with snRNA sequences, were used for snoRNA target prediction.

### Non-coding RNA annotation

The PatMatch program [59] was used to scan the genome for the presence of conserved promoter elements: *Telo*-box (AAACCCTA) and six associated permutations (AACCCTAA, ACCCTAAA, CCCTAAAC, CCTAAACC, CTAAACCC and TAAACCCT), Site II element (TGGGCY) and TATA box (TATAAA, TATATA, ATATAA, ATAAAT, TAAATA, ATATAT, TTATAA and TTATAT). Potential open reading frames (ORFs) of novel transcripts were predicted by ESTScan using Arabidopsis parameters [40]. Putative snoRNA was predicted by SnoReport (version 1.2.3) with default ‘-smart’ parameters [41]. RNA folding structures were predicted by RNAfold (ViennaRNA package 2) [60]. The Rfam database (version 12.0, http://rfam.sanger.ac.uk/) [34] was employed to eliminate the known RNA duplicates found among our novel ncRNA candidates. The putative target sites were predicted by the PLEXY program [43] for C/D box snoRNAs and by the RNAsnoop program [44] for H/ACA box snoRNAs by using default parameters.

### RNA-Seq data analysis

RNA sequencing data were obtained from various Arabidopsis tissues and plants grown in different conditions (Additional file 13: Table S7). Reads were mapped to the TAIR10 *Arabidopsis thaliana* reference genome using Bowtie2 (version 2.2.3) [61] with following command: bowtie2 –D 15 –R 2 –N 0 –L 22 –i S,1,1.5 –score –min L,-0.6,-0.6 –a –p 12 –q –x –S. Use of these parameters resulted in alignments containing up to 29 % of mismatches, to account for any post-transcriptional modification of the compared sequences. The resulting SAM file was further converted into BAM file format and sorted by SAMtools (version 1.0) [62].

### Polycistronic sno-miRNA identification

First, 500 nt flanking regions of each annotated ath-miRNA were extracted using a homemade perl script. Subsequently, the SnoReport program was used to scan these regions. If any putative snoRNA was predicted and did not overlap with mature miRNA, it was be considered a snoRNA-miRNA candidate. The analysis of homology with other organisms was carried out by using BLAST. For homologous snoRNA detection, the parameters were set as follows: E-value < 1e-5; similarity > 80 %. For homologous miRNA detection, a maximum of two mismatches were allowed.

## Availability of supporting data

The data supporting the results of this article are included within the article and its additional files.

## Additional files

Additional file 1: Table S1. List of Arabidopsis mRNA genes containing Telo-box and Site II elements in 1kb upstream of TSS. (XLSX 134 kb) Additional file 2: Figure S1. Schematic representation and RT-PCR validation of newly identified snoRNA/scaRNAs. (a) Novel snoRNA/scaRNAs are shown by grey pentagons. The negative numbers indicate the position of the Telo-box, Site II element and TATA box. The primers used for RT-PCR are underlined by black arrows. (b) RT-PCR analysis of snoRNA/scaRNA expression in 2-wk-old seedlings. Genomic DNA, G, was used as a positive control. RT+ and RT- refer to the presence and absence of reverse transcriptase, respectively. Molecular markers are shown as M. The expected RT-PCR product sizes are indicated below the lanes. (PDF 79 kb) Additional file 3: Table S2. List of three sno-miRNA genes containing TeloSII in *A.th.* (DOCX 16 kb) Additional file 4: Figure S2. 5’ and 3’ RACE mapping of the TSS and TTS in the sno-miR775 gene. Single major signals in 5’ nested RACE PCR (left) and 3’ nested RACE PCR (right) indicate the transcription start site (TSS) and transcription termination site (TTS), respectively, of the sno-miR775 gene. (PDF 50 kb) Additional file 5: Figure S3. The distribution of small RNAs on the sno-miR775 and (b) sno-miR779. Small RNA sequences were adopted from following samples: GSM575246 (WT), GSM154361 (*dcl1-7*), GSM1533542 (*dcl3*) GSM154364 (*dcl4-2*), GSM893124 (*rdr2*) and GSM575247 (*rdr6*). The visualization of reads mapping was carried out by Golden Helix GenomeBrowse® visualization tool v2.1.0. (PDF 179 kb) Additional file 6: Table S3. List of sno-miRNA candidates in rice. (DOCX 20 kb) Additional file 7: Figure S4. RT-PCR validation of sno-miRNA gene precursors in *Oryza sativa* inflorescence tissue. (a) Schematic diagram of sno-miRNA genes. The snoRNA and the miRNA are denoted by a gray pentagon and an open pentagon, respectively. The TeloSII elements and TATA box are represented by different boxes: Telo-box (open box), Site II (gray box), and TATA box (black box). The primers designed for sno-miRNA precursors and the expected sizes are indicated by arrows and solid lines. (b) RT-PCR analysis of sno-miRNA precursors expression in inflorescence tissue. RT+ and RT- refer to the presence and absence of reverse transcriptase, respectively. Molecular markers are shown as M. Genomic DNA, G, was used as a positive control. Actin protein (Os05g01600.2) is used as loading control. (PDF 347 kb) Additional file 8: Figure S5. Sequence features of sno-miRNA genes in *Oryza Sativa*. Mature miRNAs are in purple. Mature snoRNAs are in bold. C/D and H/ACA boxes are in red. Inverted repeats predicted as the 5’ and 3’ termini are underlined. (PDF 37 kb) Additional file 9: Figure S6. Schematic representation and RT-PCR validation of novel ncRNAs. (a) Schematic representation of novel ncRNAs. The ncRNAs are denoted by open dashed pentagons. The termini were examined by RNA-Seq data and EST/MPSS sequences, denoted by grey arrows. The primers designed for ncRNA precursors and the expected sizes are indicated by arrows. (b) RT-PCR analysis was carried out in 2-wk-old seedlings. RT+ and RT- refer to the presence and absence of reverse transcriptase, respectively. Molecular markers are shown as M. Genomic DNA, G, was used as a positive control. The expected RT-PCR product size is indicated below the lanes. (PDF 69 kb) Additional file 10: Table S4. List of 60 lincRNAs containing Telo-box and site II elements. (XLSX 15 kb) Additional file 11: Table S5. List of other sno-miRNA candidates without TeloSII in *A.th*. (DOCX 17 kb) Additional file 12: Table S6. List of primers and hybridization probes used in the experiments. (XLSX 16 kb) Additional file 13: Table S7. List of RNA-Seq datasets. (DOCX 41 kb)

## Abbreviations

ncRNA: non-coding RNA
miRNA: microRNA
siRNA: small interfering RNA
snoRNA: small nucleolar RNA
scaRNA: small Cajal body-specific RNA
rRNA: ribosomal RNA
snRNA: small nuclear RNA
CRE: *cis*-regulatory element
snoRNP: small nucleolar ribonucleoprotein
TE: transposable element
TSS: transcription start site
TTS: transcription termination site
