# Supplementary material for: Promoter-based identification of novel non-coding RNAs reveals the presence of dicistronic snoRNA-miRNA genes in Arabidopsis thaliana
Source: BMC Genomics. 2015 Nov 25;16:1009. doi: 10.1186/s12864-015-2221-x (PMC4660826; doi:10.1186/s12864-015-2221-x)
Supplement: Additional file 2: Figure S1. — Schematic representation and RT-PCR validation of newly identified snoRNA/scaRNAs. (a) Novel snoRNA/scaRNAs are shown by grey pentagons. The negative numbers indicate the position of the Telo-box, Site II element and TATA box. The primers used for RT-PCR are underlined by black arrows. (b) RT-PCR analysis of snoRNA/scaRNA expression in 2-wk-old seedlings. Genomic DNA, G, was used as a positive control. RT+ and RT- refer to the presence and absence of reverse transcriptase, respectively. Molecular markers are shown as M. The expected RT-PCR product sizes are indicated below the lanes. (PDF 79 kb) [file 12864_2015_2221_MOESM2_ESM.pdf]

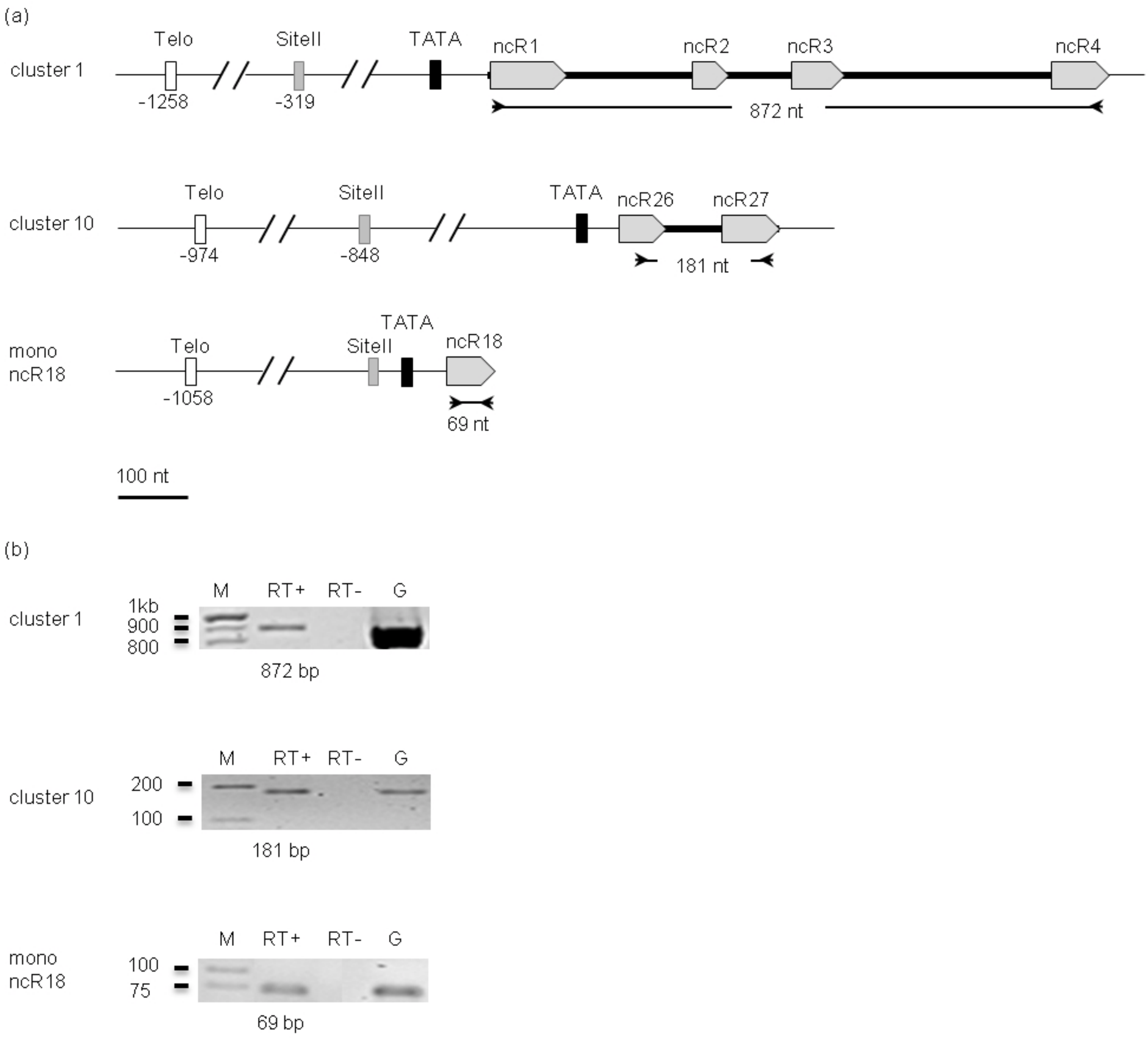

Figure S1. Schematic representation and RT-PCR validation of newly identified snoRNA/scaRNAs. (a) Novel snoRNA/scaRNAs are shown by grey pentagons. The negative numbers indicate the position of the Telo-box, Site II element and TATA box. The primers used for RT-PCR are underlined by black arrows. (b) RT-PCR analysis of snoRNA/scaRNA expression in 2-wk-old seedlings. Genomic DNA, G, was used as a positive control. RT+ and RT- refer to the presence and absence of reverse transcriptase, respectively. Molecular markers are shown as M. The expected RT-PCR product sizes are indicated below the lanes.
