# Supplementary material for: Promoter-based identification of novel non-coding RNAs reveals the presence of dicistronic snoRNA-miRNA genes in Arabidopsis thaliana
Source: BMC Genomics. 2015 Nov 25;16:1009. doi: 10.1186/s12864-015-2221-x (PMC4660826; doi:10.1186/s12864-015-2221-x)
Supplement: Additional file 3: Table S2. — List of three sno-miRNA genes containing TeloSII in A.th. (DOCX 16 kb) [file 12864_2015_2221_MOESM3_ESM.docx]

**Table S2.** List of three sno-miRNA genes containing TeloSII in *A.th.*

| **ID** | **Components** | **Coordinates** | **cDNA/EST** | **RNA-Seq** | **Homology^a^** |
| --- | --- | --- | --- | --- | --- |
| sno-miR158b | ncR100 | Chr1:20772330..20772435 | / | / | *A.ly* |
|  | miR158b | Chr1:20772273..20772292 | / | GSM575247 |  |
| sno-miR779 | snoR128 | Chr2:9560355..9560446 | AF317971 | GSM575246 | */* |
|  | snoR129 | Chr2:9560536..9560623 | / | GSM575246 |  |
|  | miR779.1 | Chr2:9560902..9560922 | / | GSM575246 |  |
|  | miR779.2 | Chr2:9560866..9560886 | / | GSM575246 |  |
| sno-miR775 | snoR775 | Chr1:29422149..29422275 | BX818024 | GSM575246 | */* |
|  | miR775 | Chr1:29422546..29422565 | BX818024 | GSM575246 |  |

^a^ Conservation analysis were performed in *Arabidopsis lyrata (A.ly), Capsella rubella (C.ru), Brassica rapa (B.ra), Medicago truncatula (M.tr)* and *Oryza sativa (O.sa).*
