# Supplementary material for: Promoter-based identification of novel non-coding RNAs reveals the presence of dicistronic snoRNA-miRNA genes in Arabidopsis thaliana
Source: BMC Genomics. 2015 Nov 25;16:1009. doi: 10.1186/s12864-015-2221-x (PMC4660826; doi:10.1186/s12864-015-2221-x)
Supplement: Additional file 4: Figure S2. — 5’ and 3’ RACE mapping of the TSS and TTS in the sno-miR775 gene. Single major signals in 5’ nested RACE PCR (left) and 3’ nested RACE PCR (right) indicate the transcription start site (TSS) and transcription termination site (TTS), respectively, of the sno-miR775 gene. (PDF 50 kb) [file 12864_2015_2221_MOESM4_ESM.pdf]

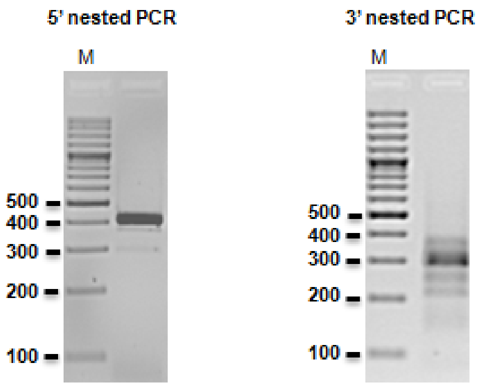

Figure S2. 5' and 3' RACE mapping of the TSS and TTS in the sno-miR775 gene. Single major signals in 5' nested RACE PCR (left) and 3' nested RACE PCR (right) indicate the transcription start site (TSS) and transcription termination site (TTS), respectively, of the sno-miR775 gene.
