# Supplementary material for: Promoter-based identification of novel non-coding RNAs reveals the presence of dicistronic snoRNA-miRNA genes in Arabidopsis thaliana
Source: BMC Genomics. 2015 Nov 25;16:1009. doi: 10.1186/s12864-015-2221-x (PMC4660826; doi:10.1186/s12864-015-2221-x)
Supplement: Additional file 5: Figure S3. — The distribution of small RNAs on the sno-miR775 and (b) sno-miR779. Small RNA sequences were adopted from following samples: GSM575246 (WT), GSM154361 (dcl1-7), GSM1533542 (dcl3) GSM154364 (dcl4-2), GSM893124 (rdr2) and GSM575247 (rdr6). The visualization of reads mapping was carried out by Golden Helix GenomeBrowse® visualization tool v2.1.0. (PDF 179 kb) [file 12864_2015_2221_MOESM5_ESM.pdf]

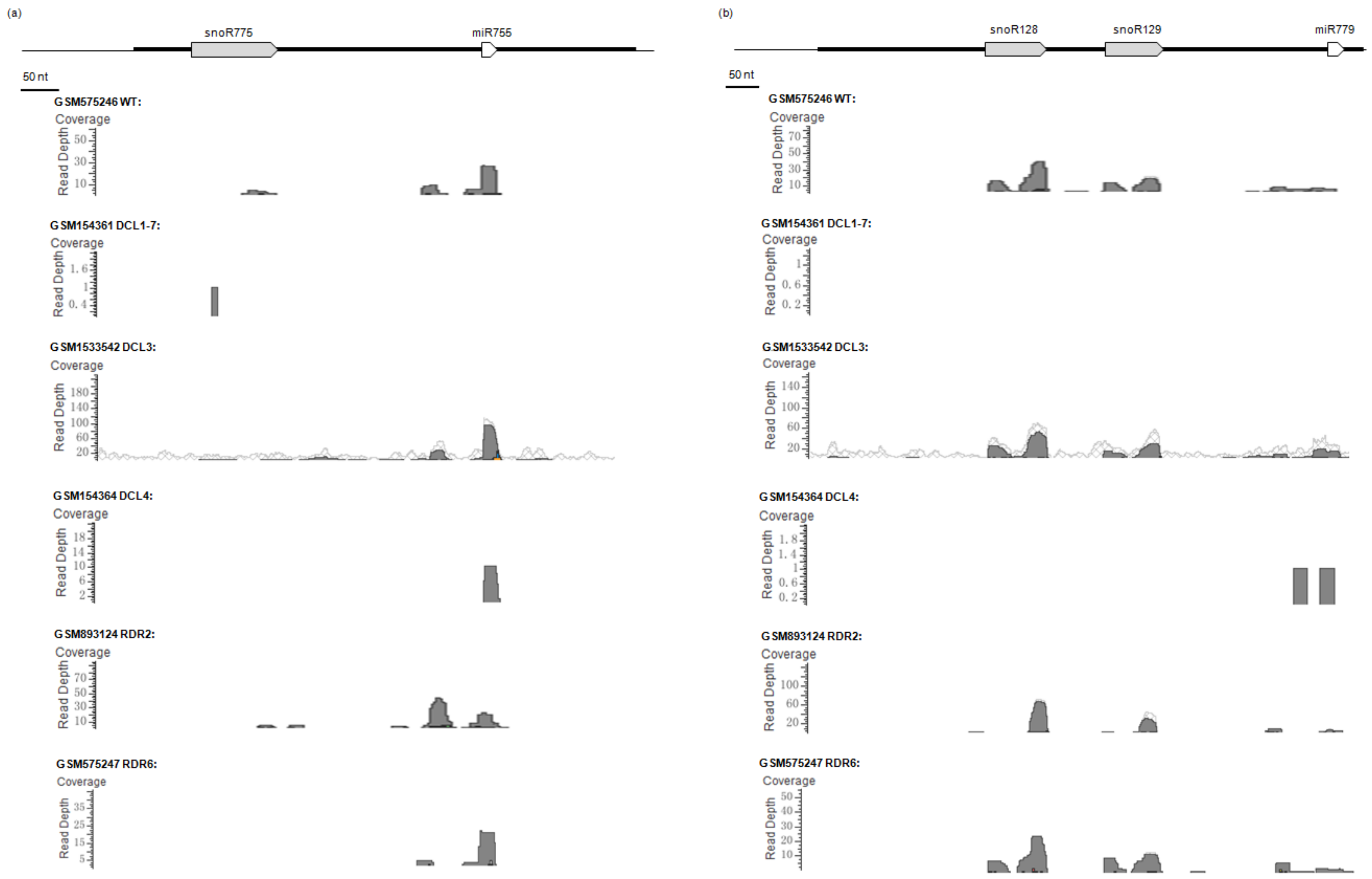


**Figure S3. The distribution of small RNAs on the sno-miR775 and (b) sno-miR779.** Small RNA sequences were adopted from following samples: GSM575246 (WT), GSM154361 (*dcl1-7*), GSM1533542 (*dcl3*) GSM154364 (*dcl4-2*), GSM893124 (*rdr2*) and GSM575247 (*rdr6*). The visualization of reads mapping was carried out using Golden Helix GenomeBrowse® visualization tool v2.1.0. The light dash lines above the solid peaks in the plots indicate multi-mapped alignments.
