# Supplementary material for: Promoter-based identification of novel non-coding RNAs reveals the presence of dicistronic snoRNA-miRNA genes in Arabidopsis thaliana
Source: BMC Genomics. 2015 Nov 25;16:1009. doi: 10.1186/s12864-015-2221-x (PMC4660826; doi:10.1186/s12864-015-2221-x)
Supplement: Additional file 6: Table S3. — List of sno-miRNA candidates in rice. (DOCX 20 kb) [file 12864_2015_2221_MOESM6_ESM.docx]

**Table S3.** List of sno-miRNA candidates in rice.

| **ID** | **Components^a^** | **Coordinates** | **cDNA/EST** | **Telo+siteII^b^** |
| --- | --- | --- | --- | --- |
| osa-sno-miR5824 | osa-MIR5824 | Chr1:1761730..1761530 | / | No |
|  | ncR200 (box C/D) | Chr1:1761913..1761814 | CI737970 |  |
| osa-sno-miR5521 | osa-MIR5521 | Chr1:2334260..2334077 | CI450839 | No |
|  | ncR201 (box H/ACA) | Chr1:2334454..2334317 | CI450839 |  |
| osa-sno-miR156c | osa-MIR156c | Chr1:4665975..4666123 | AK110797 | No |
|  | ncR202 (box C/D) | Chr1:4666195..4666267 | AK110797 |  |
|  | osa-MIR156b | Chr1:4666341..4666516 | AK110797 | No |
|  | ncR203 (box C/D) | Chr1:4666794..4666900 | AK110797 |  |
| osa-sno-miR2925 | osa-MIR2925 | Chr1:25801058..25801177 | CI280129 | No |
|  | ncR204 (box H/ACA) | Chr1:25801548..25801672 | FG952737 |  |
| osa-sno-miR167j | osa-MIR167j | Chr1:32686227..32686068 | CT834816 | No |
|  | ncR205 (box C/D) | Chr1:32686239..32686166 | CT834816 |  |
| osa-sno-miR5083 | osa-MIR5083 | Chr1:41010952..41010573 | / | Yes |
|  | snoR41^△^ | Chr1:41010843..41010763 |  |  |
|  | snoZ155^△^ | Chr1:41010655..41010579 |  |  |
| osa-sno-miR827 | osa-MIR827 | Chr2:23901284..23901168 | CT830018 | No |
|  | ncR208 (box C/D) | Chr2:23900838..23900765 | CT830018 |  |
| osa-sno-miR396 | osa-MIR396a | Chr2:34280384..34280537 | CA765692 | No |
|  | ncR209 (box C/D) | Chr2:34280406..34280476 | CA765692 |  |
| osa-sno-miR160d | osa-MIR160d | Chr3:32454988..32455129 | CK053349 | Yes |
|  | ncR210 (box C/D) | Chr3:32455542..32455629 | CI390226 |  |
| osa-sno-miR530 | osa-MIR530 | Chr4:8303694..8303538 | CK082174 | No |
|  | ncR211 (box H/ACA) | Chr4:8303648..8303528 | CK082174 |  |
| osa-sno-miR171c | osa-MIR171c | Chr4:31713570..31713472 | CI684697 | No |
|  | ncR212 (box C/D) | Chr4:31713648..31713583 | CI684697 |  |
| osa-sno-miR1425 | osa-MIR1425 | Chr5:8862149..8862250 | AK101146 | No |
|  | ncR213 (box C/D) | Chr5:8862286..8862379 | AK101146 |  |
| osa-sno-miR1850 | osa-MIR1850 | Chr5:26275339..26275207 | EU563917 | Yes |
|  | snoZ193^△^ | Chr5:26275624..26275524 | EU563917 |  |
|  | snoU83^△^ | Chr5:26275824..26275724 | EU563917 |  |
|  | snoZ191^△^ | Chr5:26275962..26275879 | EU563917 |  |
|  | snoR32^△^ | Chr5:26276162..26276058 | EU563917 |  |
| osa-sno-miR169b | osa-MIR169b | Chr6:27296035..27295908 | CI449688 | No |
|  | ncR218 (box C/D) | Chr6:27295829..27295753 | CI449688 |  |
| osa-sno-miR6250 | osa-MIR6250 | Chr6:28264615..28264740 | AK107197 | No |
|  | snoR134^△^ | Chr6:28264861..28265009 | AK107197 |  |
|  | snoU36a^△^ | Chr6:28265075..28265172 | AK107197 |  |
|  | snoZ223^△^ | Chr6:28265245..28265341 | AK107197 |  |
|  | snoZ278^△^ | Chr6:28265446..28265578 | AK107197 |  |
| osa-sno-miR166b | osa-MIR166b | Chr6:30327289..30327084 | CT858092 | No |
|  | ncR223 (box C/D) | Chr6:30327043..30326954 | CT858092 |  |
| osa-sno-miR6255 | osa-MIR6255 | Chr7:25018870..25018786 | AK288746 | No |
|  | snoR135^△^ | Chr7:25019157..25019008 | AK288746 |  |
| osa-sno-miR1858 | osa-MIR1858a | Chr9:13536611..13536810 | CV727959 | Yes |
|  | ncR226 (box H/ACA) | Chr9:13536594..13536727 | CV727959 |  |
| osa-sno-miR2118 | osa-MIR2118r | Chr11:7810397..7810223 | CK053209 | No |
|  | ncR228 (box H/ACA) | Chr11:7809956..7809808 | CK053209 |  |
| osa-sno-miR5535 | osa-MIR5535 | Chr12:12446302..12445975 | CR278335 | No |
|  | ncR230 (box H/ACA) | Chr12:12446447..12446321 | CR278335 |  |

^a^ snoRNA with triangle was identified in previous studies.

^b^ Whether contains both Telo and site II elements in 1kb upstream region
