# Supplementary material for: Promoter-based identification of novel non-coding RNAs reveals the presence of dicistronic snoRNA-miRNA genes in Arabidopsis thaliana
Source: BMC Genomics. 2015 Nov 25;16:1009. doi: 10.1186/s12864-015-2221-x (PMC4660826; doi:10.1186/s12864-015-2221-x)
Supplement: Additional file 7: Figure S4. — RT-PCR validation of sno-miRNA gene precursors in Oryza sativa inflorescence tissue. (a) Schematic diagram of sno-miRNA genes. The snoRNA and the miRNA are denoted by a gray pentagon and an open pentagon, respectively. The TeloSII elements and TATA box are represented by different boxes: Telo-box (open box), Site II (gray box), and TATA box (black box). The primers designed for sno-miRNA precursors and the expected sizes are indicated by arrows and solid lines. (b) RT-PCR analysis of sno-miRNA precursors expression in inflorescence tissue. RT+ and RT- refer to the presence and absence of reverse transcriptase, respectively. Molecular markers are shown as M. Genomic DNA, G, was used as a positive control. Actin protein (Os05g01600.2) is used as loading control. (PDF 347 kb) [file 12864_2015_2221_MOESM7_ESM.pdf]

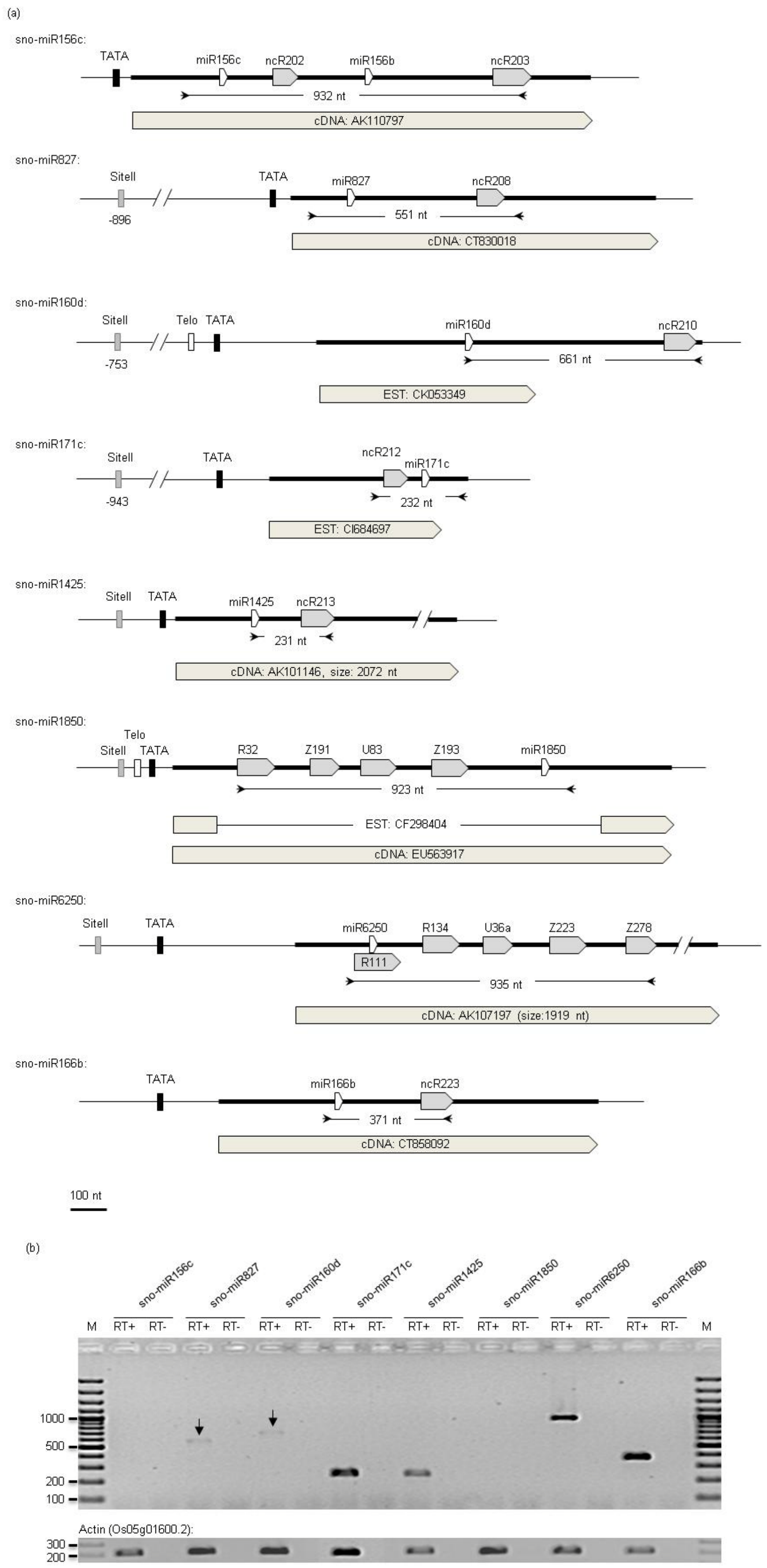

Figue S4. RT-PCR validation of sno-miRNA gene precursors in *Oryza sativa*

inflorescence tissue. (a) Schematic diagram of sno-miRNA genes. The snoRNA and the miRNA are denoted by a gray pentagon and an open pentagon, respectively. The TeloSII elements and TATA box are represented by different boxes: Telo-box (open box), Site II (gray box), and TATA box (black box). The primers designed for sno-miRNA precursors and the expected sizes are indicated by arrows and solid lines. (b) RT-PCR analysis of sno-miRNA precursors expression in inflorescence tissue. RT+ and RT- refer to the presence and absence of reverse transcriptase, respectively. Molecular markers are shown as M. Genomic DNA, G, was used as a positive control. Actin protein (Os05g01600.2) is used as loading control.
