# Supplementary material for: Promoter-based identification of novel non-coding RNAs reveals the presence of dicistronic snoRNA-miRNA genes in Arabidopsis thaliana
Source: BMC Genomics. 2015 Nov 25;16:1009. doi: 10.1186/s12864-015-2221-x (PMC4660826; doi:10.1186/s12864-015-2221-x)
Supplement: Additional file 8: Figure S5. — Sequence features of sno-miRNA genes in Oryza Sativa. Mature miRNAs are in purple. Mature snoRNAs are in bold. C/D and H/ACA boxes are in red. Inverted repeats predicted as the 5’ and 3’ termini are underlined. (PDF 37 kb) [file 12864_2015_2221_MOESM8_ESM.pdf]

>osa sno-miR827 Chr2:23901290..23900740
AACCTGTTTTGTTGCTGGTCATCTAGCTACCCGTGCATGCCTGGAGATTGGAGAATAATTGACGATGCAGCAGTCGGCTTATT
GGCTCTTGGGCACGCGTGGTTAGATGACCATCAGCAAACAAGTTCGTGAGACGCATGCATGGTTTCGTCGATTCTCTGGTTC
ATTTGAGGTGCGGCTGCAATAGGAGTTCCTTTTTGTCATGTATGCTTCTTCGATCTCTGCTCCCGTTGACCTCGATCTCGTTAT
CAGATTCGTGTTTTTCTTTTTCCCTCTCCGCCTTAAGAAGAAGCAAAGGCGGTCGCGTTCTCTTTGCGTGGCTCTGTCGTTAAC
TAGCTACGTCTTGCTGGTTAGGAGTAGTTCTGAATTTGCATTGCAGACTTGCAGTGTGTGTCTCTGAAGACGACGCTGCAAAT
CGCCGTCTCCGTGTGCTTAATTACGACAGGTTTGCAAGGAACAGCGGCTGTTGATGTTGCTGATCGTCAGCAATTCCGTGTT
TATGCGTGTCTGATTGCAGGAAAAATGAGTACTAGTAGTACCTGGTATGACGA

>osa sno-miR160d Chr3:32454988..32455660
GGGGATATGCCTGGCTCCCTGTATGCCACTCGCGTAGCTGCCAAACTCAGTTGAAACAACTGCCTTCTCCCGGCGAGATTCA
GGCATTGTGTTCGTACGTTTGGCTCTACTGCGGATGGCGTGCGAGGAGCCAAGCATGACCGTCTCTCTCTGTCTCTATCTCTC
TCTCATTGACTCATTGTGTGTGCTCACTTAAGATTACACGCTTAATTAAACCTGATAAACATGGATCATACGGTAGTTTATACTG
CAGTTTTAGTAGTCTTATTAGTTCTTATTACAATTAGTCTCTTTTAATTAGTTATGCGCTTGGTTATGGTTTCTTATTAGTTTGATG
AGTAGTTATGCTTAATACATATTGCATGCATTTTATTTTTCGCTTGTGTGTGACGCTGTCATGATTTGCAGTAAGTGAGCAAACC
TGTGTATATAGTGTGTACTAATAGTTCTCTGGTTGATTACACATCTGCATGCAGTCACTTGGTGAGCAACTTCTCTGAAAAAAAT
TATTGACAGCCTAGCTAGTTTGTTTCATGTACTGTTTGCCACCTTTTTCTGATGCATGGTAGTGCTGACTGCTGACATAAATGC
ATGCATATACAATTGTTGTCATGGTTTTCTATCTTGATCTGATTAGGAGAGGTTAGATTATGGTAGATGCATGAGCTGACCT

>osa sno-miR171c Chr4:31713690..31713430
TAGTGAGGTGAGAGTACTTGAGAGGGAGTGTATTGATTGGATTGCTGTTTTTATCTCTGATGATTTGCCTTGTTTTGCTGATCT
GAATGCTGATCGTTGGGGGATTGTTCTGCTCTCTATGTGGGAACGGGATATTGGTGCGGTTCAATCAGAAAGCTTGTGCTCC
GAAGGCGAGGGGCTCCACTCTTTGATTGAGCCGTGCCAATATCACGTCGCCTTTGCAGCTGCTCGTTTGTTTATGTAAGGATG
TTTTTTTCATTA

>osa sno-miR1425 Chr5:8862149..8862379
CTGTTGACTGCATTAGGATTCAATCCTTGCTGCTAAATGTATTGCTTATATTCAGCAATATAATGTTCAGCAGCAAGAACTGGA
TCTTAATATAGTCGATAGTGGAAGAACGGTAACATATGTGGTTTGCAGCAGGTGAGCAGGATGGGTGTGGATGATTGAATAT
CTCTGTTCAGTGTTTTCATCATCTGACTGAACACTGAATCAGCTTGCTGACGTTAGAGGTTTCAG

>_osa sno-miR1850 Chr5:26276162..26275207
AGGACGGTGAAGATCGTATCTATTGGTCTCGTTCGTCGTAACTGGCAAATTGTTTTTGCCTTGCCGACCATCCTGATATATC
ACCACCCATATTCCGAGTCCTCTGTAACCAAATCAATTTTCGATTCCTCATGAGATCAGATCTTCTTTGGTGATTTTCTTTCGTT
TGGCTCTTCTTTTGGATTCATTGTGCAGTTATATTGGCCGATGATTAGATTAAAATATAGACAAGCATATGTCTGAATTTTCAT
GTGGAAAAGCCTAATCTTCTCTATGAGGTCAGATTTATTGATGCATGTCATTGTGTTGTATTTTTCGTCATCTTTATCCTTTTCG
TTTACTTTTGAGGCCTATGATGATCAATGTGGAAACCTTTCTCTCTGAAATCTTGTGAGGACATACCCTACTACTGCCTTCCT
TCTGAGGTCTCTTATCATTGTTTGGATCTGACTAAAAATCTTATTTTGATTTTTGATATGGTTTTAAAAACTATGATTTTTTAAGA
GGTTTGATGCCGATGTGGATGTAAATTGGTTGCAAGGTCGAGTGATGAGCAAAAATGCATAGTTCAGATGATCAAACCCTAG
TGGTTATGATTACTTTGAATAAATAGTCTTTCGCTCCTAACTGACGGCCTTTACGCACCCATCTCAAAAACGGTTACTAAAGAT
TTATTTATTTTGCCATATTTGATATAATTTATGTTCTCTGATTTTGTCTATCTTAACAGCCCGTCTTTCTATTTGTGTTAAACTTGA
GCGTGAACTGATCTTGCTGTAGTGCTGCATCTGTGGATACAACAACCTACCATGAACCTACCATGATGTGATGGAGATGCGAT
GGAAAGTTGGGAGATTGGGGGAAGTTGTGTGTGAACTAAACGTGGATTGGGGCCCTGTTTAGTTCACATCAATCTTCCTCCA
AATTCCCAACTTTTCATCACATCACAATCACAT

>osa sno-miR6250 Chr6:28264615..28265578
TGTTTTTTTTCTTTTTGGTTGAATCGTCCTCTCTTGTTCTTGTTAAGGTGGGGGCACCAATATATCACCATGGTGCCAGCCATCT
CTGCGAGAAGGGGATAGATCGACGCGTCAAGAAATCGTTTCTGTTGCCATGGCTCCAGTAGGCAATACGATTGTTGCTTGCG
CACAGTTCAATCTCTTGTGCAGATCAGTTTCTTCTTCTTTGTTTTTGGGTGATCTGTTGTTTATCTGTTCGAGTTGTGTTCTTGC
ACGGTGCTAACCCCGGGCTTGTCGGATTCAAATCGCCTGAAGAAGGAAGCCCCAATGCCTGTGCTAGATCCACCATACCAT
TCAGTGTTGCAGGTCTTGATGCTTCGAGCATGTGATTGATCTGTGTCAGCGTAATGGATACATTTCAAAAATCAAATGTTTTTT
TTTGTGTTTCCTAATTCTCTTTTTCCTTGGGAGATTTGATAGCTGATGGCAGTGATTTGACATTTACCCGCGCTTCTGATTTAA
ACCGTGAAGTTAAGGGAAAAAAATAATGGTTGAATTTCTTAATATGAGCCTTCCATTCTGCTATCGTATTTCCTCTAAAAATTT
GGTATTTCTGTTTGTTAGATTTACGTTTAGATTTGGTAGATTTGTCTCTTCGGCAGTGATGATACAAACTTTCAGCTTTAAACG
CCAGTTCTGCTTCTGATAATTCATGACAGATAAAGCTGATTCAGATCTGAGCCAATTGCTGTTCTACTTTTTTTCCCATAGGAA
TAACCCTTTGCGTTAGCATCATTTGTATCTCTTCTATTAGCATATTATATCATTTGTTTGAACTAATAGCATCATCTAATAATTTTA
CCTATGATGAGCTGCCCATTGTTTAGAGGATGTAGTTGGCTCGAGTTCCTGTTTATCAGGAAGAACCTACTTGTAGCTGCTG
CATTCACATGTTATCAACGGGCTACCTGAGGTCATTAGATTA

>osa sno-miR166b Chr6:30327320..30326924
AGCAAGCAAGCAAAAGTTATCTAGCTAAAGAGGGAGCCTTTTCATTTTGAGGGGAATGTTGTCTGGCTCGGGGCTACTTTTAA
TTTCTCTCTCTTTTGATATCTTCTTTTCTCGATCTCCTAGCTTGATCTTTTGATCTCTCAAATCGATCTTAAGAAAAAGATCAGTC
AAAGAGATGAGAGTAGATGTCTGTAGATCTCGGACCAGGCTTCATTCCCCCCAAACAGAAGGGCTCCCATGCATATCGATCAT
GAGCTAGCTTATTACCCCCTGCTTTTGCCTCTTCCAAATCTGATGAACTGGGTAAGTTAACTGAATATTGCTTAACTAATCAG
CTTTTTGTTTCATCTGATGGATAGATAGGAGGAGTAGATCTTGGTGGCATGAACTGCATGAG

Figure S5. Sequence features of sno-miRNA genes in *Oryza Sativa*. Mature miRNAs are in purple. Mature snoRNAs are in bold. C/D and H/ACA boxes are in red. Inverted repeats predicted as the 5’ and 3’ termini are underlined.
