# Supplementary material for: Promoter-based identification of novel non-coding RNAs reveals the presence of dicistronic snoRNA-miRNA genes in Arabidopsis thaliana
Source: BMC Genomics. 2015 Nov 25;16:1009. doi: 10.1186/s12864-015-2221-x (PMC4660826; doi:10.1186/s12864-015-2221-x)
Supplement: Additional file 9: Figure S6. — Schematic representation and RT-PCR validation of novel ncRNAs. (a) Schematic representation of novel ncRNAs. The ncRNAs are denoted by open dashed pentagons. The termini were examined by RNA-Seq data and EST/MPSS sequences, denoted by grey arrows. The primers designed for ncRNA precursors and the expected sizes are indicated by arrows. (b) RT-PCR analysis was carried out in 2-wk-old seedlings. RT+ and RT- refer to the presence and absence of reverse transcriptase, respectively. Molecular markers are shown as M. Genomic DNA, G, was used as a positive control. The expected RT-PCR product size is indicated below the lanes. (PDF 69 kb) [file 12864_2015_2221_MOESM9_ESM.pdf]

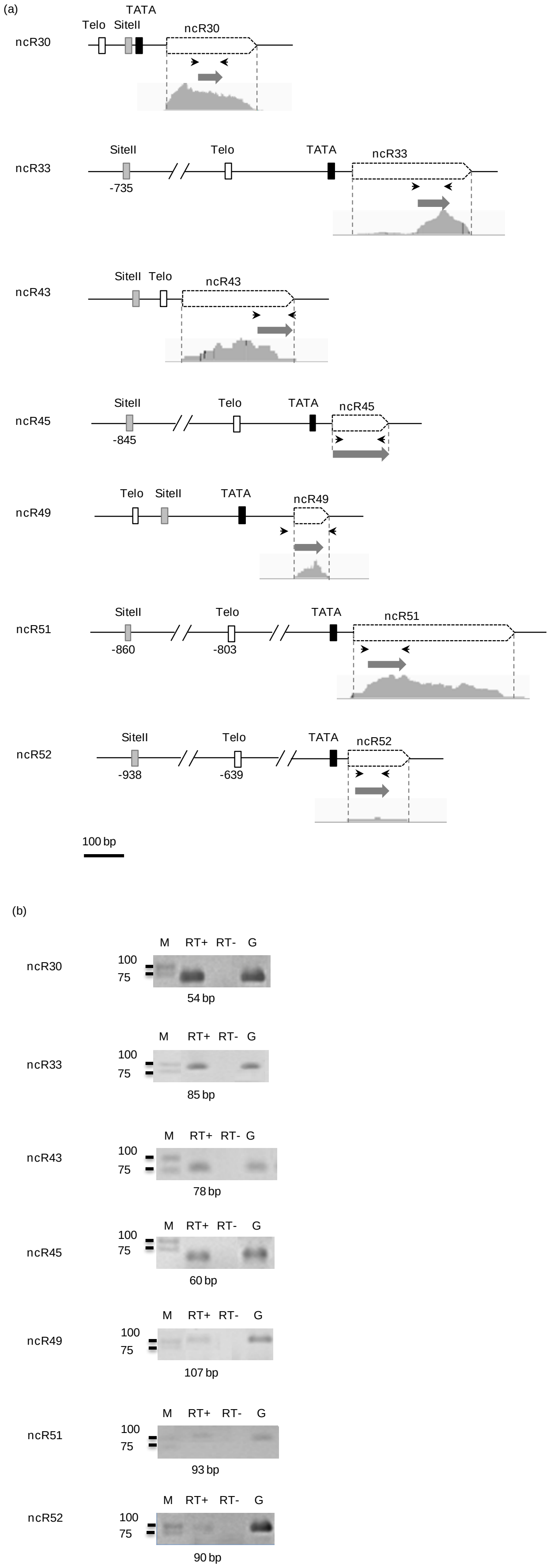

Figure S6. Schematic representation and RT-PCR validation of novel ncRNAs. (a)

Schematic representation of novel ncRNAs. The ncRNAs are denoted by open dashed pentagons. The termini were examined by RNA-Seq data and EST/MPSS sequences, denoted by grey arrows. The primers designed for ncRNA precursors and the expected sizes are indicated by arrows. (b) RT-PCR analysis was carried out in 2-week-old seedlings. RT+ and RT- refer to the presence and absence of reverse transcriptase, respectively. Molecular markers are shown as M. Genomic DNA, G, was used as a positive control. The expected RT-PCR product size is indicated below the lanes.
