# Supplementary material for: Promoter-based identification of novel non-coding RNAs reveals the presence of dicistronic snoRNA-miRNA genes in Arabidopsis thaliana
Source: BMC Genomics. 2015 Nov 25;16:1009. doi: 10.1186/s12864-015-2221-x (PMC4660826; doi:10.1186/s12864-015-2221-x)
Supplement: Additional file 11: Table S5. — List of other sno-miRNA candidates without TeloSII in A.th. (DOCX 17 kb) [file 12864_2015_2221_MOESM11_ESM.docx]

**Table S5.** List of other sno-miRNA candidates without TeloSII in *A.th.*

| **ID** | **Components** | **Coordinates** | **cDNA/EST** | **RNA-Seq** | **Tissue** |
| --- | --- | --- | --- | --- | --- |
| ath-sno-miR1 | miR850 | Chr4:7845756..7845777 | / | GSM893122 | flowers |
|  | ncR102 (box C/D) | Chr4:7846198..7846258 | EG495880 | GSM893122 | mixtures, flowers |
|  | miR863-5p | Chr4:7846644..7846664 | EG495880 | GSM896913 | mixtures, leaves |
|  | miR863-3p | Chr4:7846835..7846855 | / | GSM896913 | leaves |
| ath-sno-miR2 | ncR103 (box C/D) | Chr1:2165557..2165623 | / | GSM896913 | leaves |
|  | miR847 | Chr1:2165675..2165695 | / | GSM893121 | flowers |
| ath-sno-miR3 | ncR104 (box C/D) | Chr4:2184102..2184236 | / | SRR505744 | leaves |
|  | miR841b-5p | Chr4:2184325..2184345 | / | GSM893121 | flowers |
|  | miR841b-3p | Chr4:2184420..2184440 | / | GSM896913 | leaves |
| ath-sno-miR4 | ncR105 (box C/D) | Chr5:13622571..13622659 | / | GSM575247 | flowers |
|  | miR2937 | Chr5:13622669..13622689 | / | / | / |
| ath-sno-miR5 | miR771 | Chr3:19659402..19659381 | / | GSM893120 | flowers |
|  | ncR106 (box C/D) | Chr3:19659517..19659403 | / | GSM893124 | flowers |
|  | miR851-3p | Chr3:19659597..19659577 | / | GSM893124 | flowers |
|  | miR851-5p | Chr3:19659666..19659646 | / | GSM893115 | flowers |
