# Supplementary material for: Promoter-based identification of novel non-coding RNAs reveals the presence of dicistronic snoRNA-miRNA genes in Arabidopsis thaliana
Source: BMC Genomics. 2015 Nov 25;16:1009. doi: 10.1186/s12864-015-2221-x (PMC4660826; doi:10.1186/s12864-015-2221-x)
Supplement: Additional file 13: Table S7. — List of RNA-Seq datasets. (DOCX 41 kb) [file 12864_2015_2221_MOESM13_ESM.docx]

**Table S7.** List of RNA-Seq datasets.

| **Genotype** | **Dataset** | **Discription** | **Tissue** | **Database^a^** | **Instrument model** | **Reference** |
| --- | --- | --- | --- | --- | --- | --- |
| Treatment (Pi-) | GSM442932 rep1 | Small RNA | 17-day-old root | GEO | Illumina Genome Analyzer | [1] |
|  | GSM442934 rep2 |  |  |  |  |  |
| Treatment (Pi+) | GSM442933 rep1 | Small RNA | 17-day-old root | GEO | Illumina Genome Analyzer | [1] |
|  | GSM442935 rep2 |  |  |  |  |  |
| Treatment (Osmotic) | GSM896913 rep1 | Small RNA | 30-day-old leaf | GEO | Illumina Genome Analyzer IIx | [2] |
|  | GSM896914 rep2 |  |  |  |  |  |
| Mutant (dms4-1) | GSM456944 | Small RNA | 5-week-old flower | GEO | Illumina Genome Analyzer II | [3] |
| Mutant (rdr6) | GSM575247 | Small RNA | unopened flower buds | GEO | Illumina Genome Analyzer II | [4] |
| Mutant (nrpe1) | GSM893115 rep1 | Small RNA | immature inflorescence |  |  | [5] |
|  | GSM893116 rep2 |  |  | GEO | Illumina Genome Analyzer IIx |  |
|  | GSM893117 rep3 |  |  |  |  |  |
| Mutant (dcl1-7) | GSM154361 rep1 | Small RNA | inflorescence tissue | GEO | 454 sequencing technology | [6] |
| Mutant (dcl3) | GSM1533542 rep1 | Small RNA | inflorescence tissue | GEO | Illumina HiSeq 2000 | [7] |
| Mutant (dcl4-2) | GSM154364 | Small RNA | inflorescence tissue | GEO | 454 sequencing technology | [6] |
| Mutant (drd1) | GSM893120 | Small RNA | immature inflorescence | GEO | Illumina Genome Analyzer IIx | [5] |
| Mutant (dms3) | GSM893121 | Small RNA | immature inflorescence | GEO | Illumina Genome Analyzer IIx | [5] |
| Mutant (rdm1) | GSM893122 | Small RNA | immature inflorescence | GEO | Illumina Genome Analyzer IIx | [5] |
| Mutant (nrpd1) | GSM893123 | Small RNA | immature inflorescence | GEO | Illumina Genome Analyzer IIx | [5] |
| Mutant (rdr2) | GSM893124 | Small RNA | immature inflorescence | GEO | Illumina Genome Analyzer IIx | [5] |
| WT (flowers) | GSM869251 rep1 | Small RNA | inflorescences | GEO | Illumina HiSeq 2000 | [8] |
|  | GSM869255 rep2 |  |  |  |  |  |
| WT (Seedlings) | GSM800621 rep1 | RNA-Seq | 2-week-old seedling | GEO | Illumina Genome Analyzer II | [9] |
|  | GSM800622 rep2 |  |  |  |  |  |
| WT (roots) | SRR420813 rep1 | RNA-Seq | 13-day-old roots | SRA |  | [10] |
|  | SRR420814 rep2 |  |  |  | Illumina Genome Analyzer II |  |
|  | SRR420815 rep3 |  |  |  |  |  |
| WT (leaves) | GSM881679 rep1 | RNA-Seq | 3-week-old leaves | GEO | Illumina HiSeq 2000 | [11] |
|  | GSM881683 rep2 |  |  |  |  |  |
| WT (roots) | GSM946221 | RNA-Seq | 2-week-old roots | GEO | Illumina HiSeq 2000 | [12] |
| WT (leaves) | GSM946222 | RNA-Seq | 2-week-old leaves | GEO | Illumina HiSeq 2000 | [12] |
| WT (flowers) | GSM946223 | RNA-Seq | 5-week-old flowers | GEO | Illumina HiSeq 2000 | [12] |
| WT (flower buds) | GSM575246 | Small RNA | unopened flower buds | GEO | Illumina Genome Analyzer II | [4] |
| WT (silique) | GSM946224 | RNA-Seq | 5-week-old silique | GEO | Illumina HiSeq 2000 | [12] |
| WT (silique) | GSM385393 rep1 | Small RNA | 5-day-old post-fertilization silique | GEO | Illumina Genome Analyzer | [1] |
|  | GSM385394 rep2 |  |  |  |  |  |
|  | GSM385395 rep3 |  |  |  |  |  |
|  | GSM385396 rep4 |  |  |  |  |  |

^a^ GEO, Gene Expression Omnibus (http://www.ncbi.nlm.nih.gov/geo/); SRA: Sequence Read Archive (http://sra.dnanexus.com/).

1. Mosher R a, Melnyk CW, Kelly K a, Dunn RM, Studholme DJ, Baulcombe DC: **Uniparental expression of PolIV-dependent siRNAs in developing endosperm of Arabidopsis.** *Nature* 2009, **460**:283–286.

2. Kinoshita N, Wang H, Kasahara H, Liu J, MacPherson C, Machida Y, Kamiya Y, Hannah M a., Chua N-H: **IAA-Ala Resistant3, an Evolutionarily Conserved Target of miR167, Mediates Arabidopsis Root Architecture Changes during High Osmotic Stress**. *Plant Cell* 2012, **24**:3590–3602.

3. Kanno T, Bucher E, Daxinger L, Huettel B, Kreil DP, Breinig F, Lind M, Schmitt MJ, Simon S a, Gurazada SGR, Meyers BC, Lorkovic ZJ, Matzke AJM, Matzke M: **RNA-directed DNA methylation and plant development require an IWR1-type transcription factor.** *EMBO Rep* 2010, **11**:65–71.

4. Zheng Q, Ryvkin P, Li F, Dragomir I, Valladares O, Yang J, Cao K, Wang LS, Gregory BD: **Genome-wide Double-stranded RNA sequencing reveals the functional significance of Base-paired RNAs in Arabidopsis**. *PLoS Genet* 2010, **6**.

5. Lee TF, Gurazada SGR, Zhai J, Li S, Simon S a., Matzke M a., Chen X, Meyers BC: **RNA polymerase V-dependent small RNAs in Arabidopsis originate from small, intergenic loci including most SINE repeats**. *Epigenetics* 2012, **7**:781–795.

6. Kasschau KD, Fahlgren N, Chapman EJ, Sullivan CM, Cumbie JS, Givan S a., Carrington JC: **Genome-wide profiling and analysis of Arabidopsis siRNAs**. *PLoS Biol* 2007, **5**:0479–0493.

7. Groth M, Stroud H, Feng S, Greenberg MVC, Vashisht A a, Wohlschlegel J a, Jacobsen SE, Ausin I: **SNF2 chromatin remodeler-family proteins FRG1 and -2 are required for RNA-directed DNA methylation.** *Proc Natl Acad Sci U S A* 2014, **111**:17666–71.

8. Zhao Y, Yu Y, Zhai J, Ramachandran V, Theresa T: **HESO1, a nucleotidyl transferase in Arabidopsis, uridylates unmethylated miRNAs and siRNAs to trigger their degradation**. *Curr Biol* 2012, **22**:689–694.

9. Rogers MF, Thomas J, Reddy AS, Ben-Hur A: **SpliceGrapher: detecting patterns of alternative splicing from RNA-Seq data in the context of gene models and EST data**. *Genome Biol* 2012, **13**:R4.

10. Lan P, Li W, Schmidt W: **Complementary Proteome and Transcriptome Profiling in Phosphate-Deficient Arabidopsis Roots Reveals Multiple Levels of Gene Regulation**. *Mol Cell Proteomics* 2012:1156–1166.

11. Ausin I, Greenberg MVC, Simanshu DK, Hale CJ, Vashisht a. a., Simon S a., Lee T -f., Feng S, Espanola SD, Meyers BC, Wohlschlegel J a., Patel DJ, Jacobsen SE: **Inaugural Article: INVOLVED IN DE NOVO 2-containing complex involved in RNA-directed DNA methylation in Arabidopsis**. *Proc Natl Acad Sci* 2012, **109**:8374–8381.

12. Liu J, Jung C, Xu J, Wang H, Deng S, Bernad L, Arenas-Huertero C, Chua N-H: **Genome-wide analysis uncovers regulation of long intergenic noncoding RNAs in Arabidopsis.** *Plant Cell* 2012, **24**:4333–45.
